# Management and outcome of mechanically ventilated patients after cardiac arrest

**DOI:** 10.1186/s13054-015-0922-9

**Published:** 2015-05-08

**Authors:** Yuda Sutherasan, Oscar Peñuelas, Alfonso Muriel, Maria Vargas, Fernando Frutos-Vivar, Iole Brunetti, Konstantinos Raymondos, Davide D’Antini, Niklas Nielsen, Niall D Ferguson, Bernd W Böttiger, Arnaud W Thille, Andrew R Davies, Javier Hurtado, Fernando Rios, Carlos Apezteguía, Damian A Violi, Nahit Cakar, Marco González, Bin Du, Michael A Kuiper, Marco Antonio Soares, Younsuck Koh, Rui P Moreno, Pravin Amin, Vinko Tomicic, Luis Soto, Hans-Henrik Bülow, Antonio Anzueto, Andrés Esteban, Paolo Pelosi, For the VENTILA GROUP

**Affiliations:** Department of Medicine, Ramathibodi Hospital, Mahidol University, RAMA VI road, Bangkok, 10400 Thailand; Department of Surgical Sciences and Integrated Diagnostics IRCCS AOU San Martino-IST, Largo Rosanna Benzi 8, Genoa, 16131 Italy; Hospital Universitario Infanta Cristina and CIBER Enfermedades Respiratorias, Avenida 9 de junio, 2, 28981 Parla Madrid, Spain; Biostatistics Unit, Ramón y Cajal Institute and Research Health, IRYCIS, CIBERESP, Hospital Ramón y Cajal Ctra., Colmenar Km 9.100, 28034 Madrid, Spain; Department of Neurosciences, Odonthostomatological and Reproductive Sciences, University of Naples, “Federico II”, Naples, 80100 Italy; Hospital Universitario de Getafe and CIBER Enfermedades Respiratorias, Carretera de Toledo Km 12.500, 28905 Madrid, Spain; Anaesthesiology and Intensive Care Medicine, Medical School Hanover, 544 Carl-Neuberg-Strasse 1, D-30625 Hanover, Germany; Dipartimento di Anestesia, Rianimazione e Terapia Intensiva, Universita’ degli Studi di Foggia, Viale Pinto, 1, 71100 Foggia, Italy; Department of Anesthesia and Intensive Care, Intensive Care Unit, Helsingborg Hospital, S Vallgatan 5, 251 87 Helsingborg, Sweden; Interdepartmental Division of Critical Care Medicine, Department of Medicine, University of Toronto, University Health Network and Mount Sinai Hospital, 585 University Avenue, Toronto, M5G 2N2 ON Canada; Department of Anaesthesiology and Intensive Care Medicine, University Hospital of Cologne, Kerpener Straße 62, 50937 Köln, Germany; Cenre Hospitalier Universitaire de Poitiers, Réanimation Médicale, INSERM CIC 1402, Université de Poitiers, Poitiers, 86000 France; Department of Epidemiology and Preventive Medicine, ANZIC-RC, Monash University, Commercial Road, Melbourne, 3004 Australia; Dept. Pathophysiology, Hospital de Clínicas, Av. Italia s/n. Universidad de la Republica, Montevideo, 11600 Uruguay; Department of Intensive Care, Hospital Nacional Prof. Alejandro Posadas El Palomar, Buenos Aires, CP 1684 Argentina; Medical Staff-Critical Care, Hospital Prof. Dr. Luis Guemes, Buenos Aires, Argentina; Anesthesiology and Intensive Care, Istanbul University, Istanbul Medical Faculty, Millet cad., 34093 Istanbul, Turkey; Clínica Medellín & Universidad Pontificia Bolivariana, Medellín, Colombia; Medical ICU, Peking Union Medical College Hospital, 1 Shuai Fu Yuan, Beijing, 100730 People’s Republic of China; Department of Intensive Care, Medical Center Leeuwarden Henri Dunantweg 2, 8934 AD Leeuwarden, The Netherlands; Hospital Universitário São José, Belo Horizonte, Brazil; Department of Pulmonary and Critical Care Medicine Asan Medical Center, Univ. of Ulsan College of Medicine, 388-1 Pungnap Dong Songpa Ku Seoul, 138-736 Seoul, Republic of Korea; Unidade de Cuidados Intensivos Neurocríticos Hospital de São José Centro Hospitalarde Lisboa Central, E.P.E. R. José António Serrano, 1150-199 Lisbon, Portugal; Bombay Hospital Institute of Medical Sciences, 12 New Marine Lines, Mumbai, 400020 India; Clínica Las Lilas de Santiago, Santiago, Chile; Instituto Nacional del Tórax de Santiago, Santiago, Chile; Anaesthesiology and Intensive Care, Holbaek Hospitall, Region Zealand University of Copenhagen, Smedelundsgade, 60 4300 Holbaek, Denmark; South Texas Veterans Health Care System and University of Texas Health Science Center, 111 E 7400 Merton Minter blvd, 78229 San Antonio, TX USA

## Abstract

**Introduction:**

The aim of this study was to describe and compare the changes in ventilator management and complications over time, as well as variables associated with 28-day hospital mortality in patients receiving mechanical ventilation (MV) after cardiac arrest.

**Methods:**

We performed a secondary analysis of three prospective, observational multicenter studies conducted in 1998, 2004 and 2010 in 927 ICUs from 40 countries. We screened 18,302 patients receiving MV for more than 12 hours during a one-month-period. We included 812 patients receiving MV after cardiac arrest. We collected data on demographics, daily ventilator settings, complications during ventilation and outcomes. Multivariate logistic regression analysis was performed to calculate odds ratios, determining which variables within 24 hours of hospital admission were associated with 28-day hospital mortality and occurrence of acute respiratory distress syndrome (ARDS) and pneumonia acquired during ICU stay at 48 hours after admission.

**Results:**

Among 812 patients, 100 were included from 1998, 239 from 2004 and 473 from 2010. Ventilatory management changed over time, with decreased tidal volumes (V_T_) (1998: mean 8.9 (standard deviation (SD) 2) ml/kg actual body weight (ABW), 2010: 6.7 (SD 2) ml/kg ABW; 2004: 9 (SD 2.3) ml/kg predicted body weight (PBW), 2010: 7.95 (SD 1.7) ml/kg PBW) and increased positive end-expiratory pressure (PEEP) (1998: mean 3.5 (SD 3), 2010: 6.5 (SD 3); *P* <0.001). Patients included from 2010 had more sepsis, cardiovascular dysfunction and neurological failure, but 28-day hospital mortality was similar over time (52% in 1998, 57% in 2004 and 52% in 2010). Variables independently associated with 28-day hospital mortality were: older age, PaO_2_ <60 mmHg, cardiovascular dysfunction and less use of sedative agents. Higher V_T_, and plateau pressure with lower PEEP were associated with occurrence of ARDS and pneumonia acquired during ICU stay.

**Conclusions:**

Protective mechanical ventilation with lower V_T_ and higher PEEP is more commonly used after cardiac arrest. The incidence of pulmonary complications decreased, while other non-respiratory organ failures increased with time. The application of protective mechanical ventilation and the prevention of single and multiple organ failure may be considered to improve outcome in patients after cardiac arrest.

**Electronic supplementary material:**

The online version of this article (doi:10.1186/s13054-015-0922-9) contains supplementary material, which is available to authorized users.

## Introduction

Many studies in patients after cardiac arrest with return of spontaneous circulation (ROSC) focus on how to improve survival and neurological outcomes. Despite several interventions, such as targeted temperature management [1-4], vasopressor drugs [5], control of seizures and blood sugar level [6], poor neurological outcome and mortality are still as high as 50% [4,7,8].

However, other organ failures should be considered in addition to neurological damage. Roberts *et al*. reported that the highest cardiovascular- and respiratory-specific Sequential Organ Failure Assessment (SOFA) scores are associated with higher in-hospital mortality in 203 post-cardiac arrest patients [9], suggesting the value of hemodynamic and respiratory optimization. A recent study demonstrated that the outcomes of mechanically ventilated patients have improved over time [10]. The characteristics and the influence of ventilator settings, that is, tidal volume and positive end-expiratory pressure (PEEP), on organ failure and outcome of patients after cardiac arrest have not been previously described.

The main aim of this study was to describe and compare the changes in ventilator management and complications over time. Secondary objectives were to investigate the potential risk factors associated with 28-day hospital mortality and development of pulmonary complications, namely acute respiratory distress syndrome (ARDS) and pneumonia acquired during intensive care unit (ICU) stay, in patients without pre-existing lung injury at ICU admission.

## Methods

### Study design

We performed a secondary analysis of three prospective observational cohort studies conducted in 1998 [11], 2004 [12] and 2010 [10] on adult patients (≥18-years-old) who received mechanical ventilation for more than 12 hours, and was performed in a total of 927 ICUs in 40 countries. National coordinators recruited local investigators from eligible ICUs (see Additional file 1). In order to minimize practice changes in response to observation, only the investigator and research coordinators at each site were aware of the exact purpose and timing of the study. The research ethics board of each participating institution approved the protocol and need for informed consent was according to local rules [10-12]. Please see Additional file 1 for details of each participant institution.

### Protocol and data collection

From the 18,302 patients enrolled, we included 812 patients (4.4%) who received mechanical ventilation after ROSC post-cardiac arrest for the purpose of this analysis. The eligible patients were those receiving mechanical ventilation caused by developing sudden cessation of cardiopulmonary function.

We collected data on baseline characteristics and blood gas measurements at ICU admission, daily ventilator settings, clinical management, and blood gas measurements, characteristics and observed complications while patients were ventilated or up to day 28. We also collected data on ICU, in-hospital and 28-day mortality and length of stay outcomes. Detailed descriptions of the variables collected, along with their definitions have previously been published [10-12]. In brief, complications arising during the course of the mechanical ventilation was defined as ARDS, pneumonia, sepsis and/or multiorgan failure (cardiovascular, respiratory, renal, hepatic and hematologic, defined as a score higher than two points on the SOFA scale. Pneumonia acquired during ICU stay was defined by modifying Centers for Disease Control and Prevention criteria which require the presence of a new radiographic infiltrate persistent for 48 hours or more plus a body temperature of more than 38.5°C or less than 35.0°C, a leukocyte count of more than 10,000/μL or less than 3,000/μL, purulent sputum or change in character of sputum, or isolation of pathogenic bacteria from an endotracheal aspirate [11].

In the 1998 cohort, data on height and Glasgow Coma Score (GCS) were not collected; therefore no data regarding tidal volume/kg predicted body weight (PBW) were available in that group. The use of neuromuscular blocking agents, sedatives and analgesic drugs was recorded daily for 28 days when the drugs were given daily for three or more hours. The onset of weaning was the time point when the physician considered the patient ready for spontaneous ventilation. Weaning was categorized as a trial of spontaneous breathing and gradual reduction in the level of ventilator support. We recorded date of extubation, date of any reintubation and tracheostomy, if and when performed. Patients were prospectively followed until hospital discharge.

### Statistical analysis

Data are expressed as mean (standard deviation), median (interquartile range) and absolute and relative frequencies, as appropriate. One-way analysis of variance (ANOVA) were used to compare continuous variables, and chi-square tests were used for categorical variables. We rejected the null hypothesis of no difference among cohorts at a nominal significance level of 0.05.

Multivariate logistic regression analysis (backward stepwise) was performed to calculate odds ratios determining which variables within 24 hours of hospital admission were associated with 28-day hospital mortality. The variables with a *P* value less than 0.1 in univariate analysis were included in multivariate analysis. Variables considered for inclusion in multivariate analysis associated with 28-day mortality were age, PaO_2_, arterial pH (pHa), use of sedative agents, cardiovascular dysfunction and renal failure during the first 24 hours of mechanical ventilation.

For the purpose of the analysis, we categorized pHa as the following: pHa <7.35, pHa 7.35 to 7.45 and pHa >7.45, according to the normal pHa range, which is 7.35 to 7.45. PaO_2_ was categorized as the following: PaO_2_ <60 mmHg, PaO_2_ 60 to 300 mmHg and PaO_2_ ≥300 mmHg, according to recent publications which demonstrated that PaO_2_ <60 mmHg and PaO_2_ ≥300 mmHg were independently associated with in-hospital mortality [13-15]. We did not include GCS in the multivariate analysis because during mechanical ventilation with sedation, the GCS is unreliable. In addition, GCS data was not collected in 1998. Odds ratios with 95% confidence intervals were calculated for statistically significant variables to determine independent predictors of mortality. These analyses were performed using SPSS version 16.0, SPSS for Windows, SPSS Inc., Chicago, USA.

The development of pulmonary complications, namely ARDS and pneumonia acquired during ICU stay, in patients without pre-existing lung injury at ICU admission were collected. We also performed multivariate logistic regression analysis to determine which variables within 24 hours of hospital admission were associated with the occurrence of ARDS and pneumonia acquired during ICU stay at 48 hours after admission. We excluded patients with diagnosed ARDS at admission. The variables considered for inclusion in the analysis were age, pHa, plateau pressure, PaO_2_ and sepsis during the first 24 hours of hospital admission.

## Results

### Characteristics of included patients and management during mechanical ventilation

In Table 1, baseline characteristics between the three cohorts are shown. Baseline characteristics including age, body mass index, gender and Simplified Acute Physiology Score (SAPS) were not different across the cohort time periods. At admission, the most significant difference was the lower GCS in patients included in 2010 versus patients included in 2004 (in 1998 this variable was not registered).

**Table 1:** **Baseline characteristics and management during mechanical ventilation of included patients**

	**Cohort 1998**	**Cohort 2004**	**Cohort 2010**	***P***
	**(N = 100)**	**(N = 239)**	**(N = 473)**	
Age, years, mean (SD)	66 (14)	63 (16)	63 (16)	0.261
Female, n (%)	37 (37)	90 (38)	174 (37)	0.966
Body mass index, kg/cm^2^, mean (SD)	na	27 (8)	27 (7)	0.754
SAPS II, points, mean (SD)	61 (19)	56 (20)	59 (20)	0.060
Glasgow Coma Score at admission, median (IQR)	na	6 (3-15)	3 (3-8)	<0.001
Arterial blood gases at admission				
pHa, mean (SD)	7.17 (0.09)	7.23 (0.20)	7.23 (0.18)	0.003
PaCO_2_, mmHg, mean (SD)	50 (13)	48 (22)	50 (23)	0.733
Ratio PaO_2_ to FiO_2_, mmHg, mean (SD)	249 (78)	233 (116)	221 (186)	0.367
Ventilatory settings during mechanical ventilation				
Tidal volume, ml/kg ABW, mean (SD)	8.9 (2)	7.4 (2)	6.7 (2)	<0.001
Tidal volume/kg PBW mean (SD)	na	9.04 (2.3)	7.95 (1.7)	<0.001
Respiratory rate, bpm, mean (SD)	17 (4)	18 (6)	19 (6)	<0.001
PEEP, cmH_2_O, mean (SD)	3.5 (3)	4.8 (4)	6.5 (3)	<0.001
Peak pressure, cmH_2_O, mean (SD)	29.1 (7.5)	27.1 (7.9)	24.1 (7.9)	<0.001
Plateau pressure, cmH_2_O, mean (SD)	22.7 (3.7)	21.5 (6.5)	19.5 (6.3)	<0.001
PaCO_2,_ , mmHg, mean (SD)	37.3 (7.4)	38.8 (10.4)	39.8 (11.7)	<0.001
pHa, mean (SD)	7.41 (0.08)	7.39 (0.1)	7.39 (0.1)	<0.001
Ratio PaO_2_ to FiO_2_, mmHg, mean (SD)	238 (95)	242 (95)	252 (114)	<0.05
Sedation, n (%)	50 (50)	175 (73)	332 (70)	<0.001
Analgesia, n (%)	20 (20)	na	272 (58)	<0.001
Neuromuscular blocking, n (%)	8 (8)	29 (12)	99 (21)	<0.001

ABW, actual body weight; IQR, interquartile range; na, no data available, PCV, Pressure controlled ventilation; PEEP, Positive end-expiratory pressure; pHa, arterial pH; SAPS, Simplified Acute Physiology Score; SD, standard deviation.

**Figure 1:**
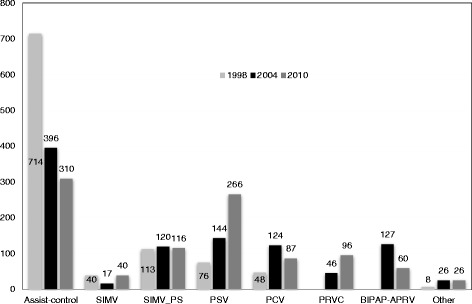
Mode of ventilation and days of use per 1,000 days of invasive mechanical ventilation from 1998, 2004 and 2010. Days during weaning from mechanical ventilation process are excluded (light gray square symbol = 1998, black square symbol = 2004 and dark gray square symbol = 2010)*. SIMV, synchronized intermittent mandatory ventilation; SIMV_PS, synchronized intermittent mandatory ventilation with pressure support; PSV, pressure support ventilation; PCV, pressure control ventilation; PRVC, pressure regulated volume control ventilation; APRV, airway pressure release ventilation; BIPAP, biphasic positive airway pressure. *Among three years, days of use per 1,000 days of invasive mechanical ventilation in each mode of ventilation are statistically significant difference (*P* <0.001).

As shown in Figure 1, the mode of mechanical ventilation, expressed as days of use per 1,000 days of invasive mechanical ventilation, changed over time with a significant increase of pressure support ventilation (PSV) and pressure regulated volume control (PRVC), and a significant decrease of other considered modes. Among ventilation settings over the years we found a significant reduction in tidal volume, peak and plateau pressure, and a significant increase of respiratory rate, PEEP and PaCO_2_. Sedation, analgesia and neuromuscular blocking were frequently used in 2010 (Table 1). At 24 hours after ICU admission, in patients with ARDS compared to those without ARDS at ICU admission, tidal volume and respiratory rate were similar (7.3 (standard deviation (SD) 1.8) ml/kg actual body weight (ABW) versus 7.5 (SD 2) ml/kg ABW, *P* = 0.613, and 18.1 (SD 5.9) rate/min versus 17.7 (SD 5.5) rate/min, *P* = 0.658), while applied PEEP was higher (7.3 (SD 4.5) cmH_2_O versus 5.2 (SD 3.1) cmH_2_O, *P* = 0.000).These results mentioned overall patients (including 1998, 2004 and 2010).

### Complications during mechanical ventilation

As shown in Table 2, the incidence of pneumonia acquired during ICU stay decreased from 13% in 1998 to 4% in 2010 (*P* = 0.001). In the meantime, other non-respiratory organ failures like sepsis, cardiovascular dysfunction, neurological and hepatic failure significantly increased.

**Table 2:** **Comparison of complications emerged over the course of mechanical ventilation**

	**Cohort 1998**	**Cohort 2004**	**Cohort 2010**	***P***
	**(N = 100)**	**(N = 239)**	**(N = 473)**	
Acute respiratory distress syndrome, n (%)	4 (4)	7 (3)	31 (7)	0.102
Acquired intensive care unit pneumonia, n (%)	13 (13)	14 (6)	18 (4)	0.001
Sepsis, n (%)	3 (3)	6 (6.5)	89 (19)	<0.001
Barotrauma, n (%)	2 (2)	6 (3)	7 (2)	0.62
Cardiovascular failure, n (%)	25 (25)	46 (19)	229 (48)	<0.001
Renal failure, n (%)	20 (20)	60 (25)	140 (30)	0.104
Hepatic failure, n (%)	2 (2)	30 (13)	24 (5)	<0.001
Hematological failure, n (%)	11 (11)	17 (7)	31 (7)	0.296
Neurological failure^a^ Glasgow coma scale, median (IQR)	na	4 (3-10)	3 (3–6)	<0.001

^a^Lowest Glasgow Coma Scale during the ventilatory support.

na, no data available; IQR, interquartile range.

### Withdrawal from mechanical ventilation

Table 3 demonstrates the characteristics of variables related to the weaning process across the three cohort time periods. The percentage of patients who were weaned and extubated was similar over time (47% in 1998, 44% in 2004 and 45% in 2010; *P* = 0.856). Among weaning methods, spontaneous breathing trial was more commonly used than gradual reduction of ventilator support. PSV was mostly used among gradual reduction of support methods, and its use tended to increase (12.5% in 1998, 78% in 2004 and 38% in 2010). In the spontaneous breathing trial group, the most common method was low-level PSV. Tracheostomy was performed in 13.8% of patients overall, and did not change significantly over time.

**Table 3:** **Comparison of variables related to weaning process**

	**Cohort 1998**	**Cohort 2004**	**Cohort 2010**	***P***
	**(N = 100)**	**(N = 239)**	**(N = 473)**	
Accidental extubation, n (%)^a^	3 (3)	6 (3)	29 (6)	0.062
Reintubation, %	67	33	14	0.074
Patients weaned and scheduled extubated, n (%)	47 (47)	104 (44)	211 (45)	0.856
Method for first attempt				
Spontaneous breathing trial, n (%)	33/47 (70)	71/104 (68)	154/211(73)	0.675
T-piece, %	48.5	38	36	0.022
CPAP, %	6	34	24	
Low level pressure support, %	42	27	40	
Other, %	3	1	0	
Gradual reduction of support, n (%)	14/47 (30)	33/104 (32)	57/211 (27)	0.675
Pressure support, %	14	61	89	<0.001
SIMV, %	29	6	0	
SIMV-PS, %	50	18	9	
Other, %	7	15	2	
Failure of first weaning attempt, n (%)	24/47 (51)	45/104 (43)	95/211 (45)	0.667
Method for second attempt				
Spontaneous breathing trial, n (%)	21 (87.5)	10 (22)	59 (62)	<0.001
T-piece, %	67	40	36	0.049
CPAP, %	5	20	34	
Low level pressure support, %	24	40	30	
Other, %	5	0	0	
Gradual reduction of support, n (%)	3 (12.5)	35 (78)	36 (38)	<0.001
Pressure support, %	0	66	94	<0.001
SIMV, %	0	6	0	
SIMV-PS, %	100	14	3	
Other, %	0	14	3	
Reintubation after scheduled extubation, %	11	7	11	0.426
Tracheotomy, n (%)^a^	12 (12)	30 (13)	66 (14.5)	0.758

^a^Excluded patients with prior tracheostomy: 1 patient in 1998, 7 patients in 2004 and 18 patients in 2010.

SIMV, synchronized intermittent mandatory ventilation; SIMV-PS, synchronized intermittent mandatory ventilation with pressure support; PSV, pressure support ventilation; CPAP, continuous positive airway pressure.

### Outcomes

We observed significant differences in the duration of ventilatory support over time, with a longer duration of mechanical ventilation in the most recent study of 2010 (Table 4). There were no differences in length of stay in the ICU or in the hospital (Table 4).

**Table 4:** **Comparison of outcomes**

	**Cohort 1998**	**Cohort 2004**	**Cohort 2010**	***P***
	**(N = 100)**	**(N = 239)**	**(N = 473)**	
Days of mechanical ventilation, median (IQR)^a^	4 (3–7)	5 (3–9)	6 (4–10)	<0.001
Length of stay in the intensive care unit, days, median (IQR)	7 (3–11)	6 (4–12)	6 (3–12)	0.925
Length of stay in the hospital, days, median (IQR)	14 (7–27)	13 (6–24)	12 (6–26)	0.934
Mortality in the intensive care unit, n (%)	44 (44)	115 (48)	223 (49)	0.785
Mortality at day 28, n (%)	52 (52)	137 (57)	246 (52)	0.384
Mortality in the hospital, n (%)	57 (57)	143 (60)	259 (55)	0.434

^a^Including time devoted to weaning from mechanical ventilation. IQR: interquartile range.

There was no difference in 28-day hospital mortality over time (52% in 1998, 57% in 2004 and 52% in 2010 (Table 4).

### Factors associated with 28-day hospital mortality

Table 5 shows the univariate and logistic regression analysis for 28-day hospital mortality of cardiac arrest patients.

**Table 5:** **Univariate and logistic regression analysis for 28-day mortality of cardiac arrest patients**

**Variable**	**Univariate analysis Odds ratio (95% CI)**	***P***	**Logistic regression Odds ratio (95% CI)**	***P***
Age, years^a^	1.02 (1.01–1.03)	0.002	1.01 (1.00–1.03)	0.010
SAPS II score, points^a^	1.03 (1.02–1.03)	<0.001		
Glasgow Coma Scale, points^b^	0.92 (0.88–0.95)	<0.001		
PaO_2_ 60–300 mmHg^b^	1 (reference)		1 (reference)	
PaO_2_ <60 mmHg	2.23 (1.05–4.72)	0.036	2.71 (1.06–6.95)	0.038
PaO_2_ ≥300 mmHg	1.19 (0.76–1.85)	0.444	0.89 (0.54–1.46)	0.640
pHa 7.35–7.45^b^	1 (reference)		1 (reference)	
Acidosis (pHa <7.35)	1.48 (1.07–2.04)	0.017	1.40 (0.98–2.02)	0.068
Alkalosis (pHa >7.45)	1.07 (0.67–1.71)	0.770	1.20 (0.71–2.02)	0.491
PaCO2 35–45 mmHg^b^	1 (reference)			
PaCO2 <35 mmHg	1.20 (0.86–1.68)	0.277		
PaCO2 >45 mmHg	0.94 (0.70–1.41)	0.973		
Tidal Volume/PBWml/kg^b^				
Tidal Volume/PBW 6–8 ml/kg	1 (reference)			
Tidal Volume/PBW <6 ml/kg	1.01 (0.51–2.02)	0.975		
Tidal Volume/PBW >8 ml/kg	0.76 ( 0.55–1.06)	0.111		
PEEP cmH_2_O^b^				
PEEP 6–8 cmH_2_O	1 (reference)			
PEEP <6 cmH_2_O	1.35 (0.94–1.95)	0.100		
PEEP >8 cmH_2_0	0.86 (0.52–1.42)	0.556		
Pplat cmH_2_O^b^				
Pplat 28–30 cmH_2_O	1 (reference)			
Pplat <28 cmH_2_O	0.58 (0.28–1.22)	0.149		
Pplat >30 cmH_2_O	0.64 (0.22–1.89)	0.421		
Use of sedative drugs^b^	0.61 (0.46–0.81)	0.001	0.51 (0.36–0.72)	0.000
Cardiovascular failure/shock (yes/no)^b,c^	1.53 (1.15–2.03)	<0.001	1.65 (1.17–2.32)	0.004
ARDS (yes/no)^b,c^	3.14 (1.41–6.97)	0.005		
Renal failure (yes/no)^b,c^	1.35 (0 .95–1.91)	0.095	1.34 (0.91–1.95)	0.135
Hepatic failure (yes/no)^b,c^	1.20 (0.72–2.00)	0.483		
Sepsis (yes/no)^b,c^	1.38 (0.88–2.18)	0.163		
Hematologic failure (yes/no)^b,c^	1.05 (0.51–2.17)	0.885		

SAPS, Simplified Acute Physiology Score; PBW, predicted body weight; ml, milliliters; kg, kilograms PEEP, positive end-expiratory pressure; Pplat, plateau pressure; pHa, arterial pH; ARDS, acute respiratory distress syndrome; PaO_2_, partial pressure of oxygen in arterial blood; PaCO_2_, partial pressure of carbon dioxide in arterial blood; CI, confidence interval.

^a^Age and SAPS score were collected as baseline characteristics, ^b^Values within 24 hours from admission, ^c^the absence of organ failure as the reference value.

In the multivariate analysis, older age, PaO_2_ <60 mmHg, less use of sedative drugs and the presence of cardiovascular dysfunction within 24 hours from hospital admission were found to be associated with 28-day hospital mortality (odds ratio 1.01, 95% confidence interval 1.00 to 1.03; odds ratio 2.71, 95% confidence interval 1.06 to 6.95; odds ratio 0.51, 95% confidence interval 0.36 to 0.72; and odds ratio 1.65, 95% confidence interval 1.17 to 2.32, respectively).

### Factors associated with acute respiratory distress syndrome and ICU-acquired pneumonia

At multivariate analysis, in patients without lung injury at admission, the potential risk factor for the development of ARDS 48 hours after ICU stay was higher plateau pressure (odds ratio 1.12, 95% confidence interval 1.04 to 1.21), while those associated with ICU pneumonia acquired during ICU stay were higher tidal volume and lower applied PEEP levels (odds ratio 1.003, 95% confidence interval 1.0003 to 1.01; and odds ratio 0.89, 95% confidence interval 0.80 to 0.99, respectively).

## Discussion

In this large retrospective analysis of prospective observational cohort, we described the evolution of ventilator management, and the occurrence of pulmonary and other non-respiratory organ failure over time. Furthermore, we investigated variables associated with 28-day hospital mortality and the occurrence of ARDS and/or pneumonia acquired during ICU stay among cardiac arrest patients undergoing mechanical ventilation. We found that: the use of protective and assisted mechanical ventilation increased from 1998 to 2010; pulmonary complications decreased, while cardiovascular and neurological complications, and sepsis increased with years; independent risk factors for 28-day hospital mortality were older age, PaO_2_ <60 mmHg, less use of sedative drugs and the presence of cardiovascular dysfunction at 24 hours after ICU admission; and in patients without lung injury at ICU admission, higher tidal volume, higher plateau pressure and lower PEEP in the first 24 hours were independent potential risk factors for developing ARDS or pneumonia acquired during ICU stay.

To our knowledge, this is the first study describing ventilator management in a large sample of patients after cardiac arrest undergoing mechanical ventilation in ICU. Our results show that protective mechanical ventilation is increasingly used among patients after cardiac arrest. The implementation of protective mechanical ventilation was associated with a progressive reduction in pneumonia acquired during ICU stay over time, and a lower incidence of ARDS than that reported in mechanically ventilated patients [16,17]. Similar changes in ventilation pattern have recently been shown in a general population of critically ill patients, associated with a reduction of development of ARDS [10,18]. Protective ventilation with low tidal volume has been shown to be associated with a reduction in respiratory failure and mortality in non-ARDS lung patients [19,20], and postoperative complications after surgery [21,22]. In donors, protective mechanical ventilation increased the number of lungs eligible to be harvested compared to traditional mechanical ventilation [23]. The application of PEEP ranging from 5 to 8 cmH_2_O in non-hypoxemic patients decreased the incidence of ventilator-associated pneumonia [24]. Moreover, protocols aimed to prevent ventilator-associated pneumonia have been more widely implemented in recent years [25,26].

On the other hand, we observed an increased incidence of non-pulmonary organ failure (sepsis, cardiovascular dysfunction and neurological failure) over time, which may increase the duration of mechanical ventilation. The increase in non-pulmonary complications may be explained by the implementation of targeted temperature management protocols, or by higher incidence of aspiration, and thus sicker patients [7], which might predispose to infection and consequent multiple organ failure [27]. The significant differences in the duration of ventilatory support over time, with a longer duration in the most recent study of 2010, is probably because of the implementation of targeted temperature management protocols, and thus longer sedation.

A previous study demonstrated that changes in mechanical ventilation practice were associated with a significant decrease in mortality [10]. In post-cardiac arrest patients, despite the introduction of temperature management, percutaneous coronary intervention and standard operating procedures, we did not observe any change in mortality over the years, likely due to the balance between decreased pulmonary and increased extra-pulmonary incidence of complication. We also found that the main independent predictors of 28-day in-hospital mortality were older age, PaO_2_ <60 mmHg, use of sedative drug, and cardiovascular dysfunction within 24 hours from admission, in line with previous reports [14,15].

In the present study, the analysis by logistic regression demonstrated that PaO_2_ <60 mmHg is a predictor of 28-day hospital mortality. This result is different from previous meta-analysis showing that not only hypoxemia but also hyperoxemia are associated with higher in-hospital mortality [28]. The effects of high oxygen tension to increase neuronal damage after cardiac arrest are conflicting [13,14,29-31]. We also found that higher or lower PaCO_2_ level had no detectable association with mortality. This was different from previous studies showing that hypocarbia defined by PaCO_2_ <35 mmHg was associated with higher in-hospital mortality [28,32], while hypercapnia defined by PaCO_2_ >45 mmHg was associated with better outcome [33,34].

Use of sedative drugs was associated with 28-day mortality in this cohort. This finding is in contrast to other studies showing that sedation protocols did not affect mortality in a general population of critically ill patients [35]. We have no completed value of GCS for the three cohort years. The lower GCS on admission in the most recent cohort would indicate more severe brain injury, and thus a lower need for sedation and a higher risk of death. On the other hand, our data suggest that higher sedation in the early phase after cardiac arrest might promote less secondary brain injury and better implementation of protective mechanical ventilation. Furthermore, the use of sedative drugs may be related to the implementation of therapeutic hypothermia, which was associated with the improvement of outcome in ROSC patients [7,8].

In Table 1, 26% (63 out of 239) of the patients in the 2004 study had a GCS of 15. The study population included patients who developed cardiac arrest and needed mechanical ventilation due to sudden and unexpected cessation of cardiopulmonary functions in any rhythms (referred to any rhythms that cause cessation of cardiopulmonary functions i.e. Pulseless electrical activity, asystole, ventricular fibrillation and ventricular tachycardia); this is not the population included in target temperature management studies, therefore we expect that a higher percentage of patients awake upon arrival in ICU. Our study is comparable with the study by Gold *et al*. [36] on patients with out-of-hospital cardiac arrest of any rhythm, which demonstrated that of the 185 survivors, 96 patients (50%) were sufficiently awake upon arrival to the ICU so they did not meet the targeted temperature management protocol inclusion criteria, but data on GCS were not reported.

In our study, we evaluated the potential independent risk factors for development of pulmonary complications in patients without pre-existing lung injury at ICU admission. We found that higher tidal volume and higher plateau pressure with lower PEEP were associated with occurrence of lung worsening during ICU stay. These findings are in line with those reported in patients without lung injury in the perioperative period [19-22], and in the ICU [19], showing that protective ventilation by low tidal volume and plateau pressure <20 cmH_2_O resulted in a decreased incidence of pulmonary complications after initiation of mechanical ventilation [19-22,37]. We also found that tidal volume was similar, while PEEP was slightly higher in patients with ARDS compared to those without ARDS at ICU admission. This suggests that protective ventilation should include lower tidal volume than actually used in daily clinical practice in cardiac arrest patients with ARDS.

The use of controlled mechanical ventilation decreased, while pressure support increased with years. The use of assisted ventilation may be associated with potential advantages like the use of less sedative agents [38-40], better hemodynamic stability [39,40], less atrophy of respiratory muscles [41] and ventilator-associated lung injury [42,43]. The rate of tracheostomy was 12 to 14.5% higher than that reported in a general population of critically ill patients (11%) [12], and comparable to that reported in neurological patients (13%) [44]. This might be explained by the possible occurrence of residual neurological deficits in cardiac arrest patients due brain hypoxia impairing cough, swallowing and secretion clearance [45].

Our study has some limitations. First, this was a *post-hoc* analysis of previously collected available data, where the statistically significant predictors of mortality might have been influenced by undefined confounding factors, that is, site, cause and initial rhythm of the cardiac arrest. A statistical *post-hoc* analysis on 28-day hospital mortality was performed to assess the power of mortality variation among the years, and showed a power of less than 50%, so the variation of mortality over years should be interpreted with caution. Second, our study focused on the details related to mechanical ventilation. Thus, we did not record possible implementation of targeted temperature management, including the details related to the causes of cardiac arrest [46]. However, a recent study showed that moderate hypothermia did not affect mortality compared to mild hypothermia [4]. Third, although the GCS was found to be associated with 28-day mortality in the univariate analysis, we did not include GCS in the multivariate analysis. Since the evaluation of GCS is not reliable within the first 72 hours during mechanical ventilation with sedation, and data were not collected in 1998. The lower GCS on admission in the most recent cohort would indicate more severe brain injury, and thus lower need for sedation and higher risk of death.

Fourth, we also did not have access to cardiac arrest-related variables, like whether the arrest was witnessed or not, whether bystander cardiopulmonary resuscitation was performed, initial presenting rhythm and the time of resuscitation commencement or return of spontaneous circulation. Fifth, in the multivariate logistic regression analysis defining risk factors associated with 28-day hospital mortality and the development of ARDS and/or pneumonia acquired in ICU, we used data collected within 24 hours after admission, therefore there were very few missing data. Nevertheless, we used variables regardless of the different years, which might have been affected by the change of clinical management, and the number of patients with development of ARDS and/or pneumonia acquired in ICU is a small portion of the whole population (5% of the total population). For this reason, the results of multivariate analysis should be interpreted with caution.

## Conclusions

Protective mechanical ventilation with lower tidal volume and higher PEEP is more commonly used after cardiac arrest. The incidence of pulmonary complications decreased, while other non-respiratory organ failures increased with time. The application of protective mechanical ventilation and the prevention of single and multiple organ failure may be considered to improve outcome in patients after cardiac arrest.

## Key messages

- The use of protective mechanical ventilation in patients after cardiac arrest increased from 1998 to 2010, and is associated with a decrease in pulmonary complications.
- Variables independently associated with 28-day in-hospital mortality were: older age, PaO_2_ <60 mmHg, cardiovascular dysfunction and less use of sedative agents.
- The application of protective mechanical ventilation and the prevention of single and multiple organ failures may be considered to improve outcome in patients after cardiac arrest.

## Additional file

Additional file 1:
**Participants in three international cohort studies on mechanical ventilation (1998, 2004 and 2010).** National coordinators recruited local investigators from eligible intensive care units. The research ethics board of each participating institution approved the protocol and need for informed consent was according to local rules.

## Abbreviations

ABW: Actual body weight
ARDS: Acute respiratory distress syndrome
pHa: Arterial pH
GCS: Glasgow Coma Score
ICU: Intensive care unit
PBW: Predicted body weight
PEEP: Positive end-expiratory pressure
PRVC: Pressure regulated volume control
PSV: Pressure support ventilation
ROSC: Return of spontaneous circulation
SAPS: Simplified acute physiology score
SOFA: Sequential organ failure assessment score
SD: standard deviation
