# Supplementary material for: Management and outcome of mechanically ventilated patients after cardiac arrest
Source: Crit Care. 2015 May 8;19(1):215. doi: 10.1186/s13054-015-0922-9 (PMC4457998; doi:10.1186/s13054-015-0922-9)
Supplement: Additional file 1: — Participants in three international cohort studies on mechanical ventilation (1998, 2004 and 2010). National coordinators recruited local investigators from eligible intensive care units. The research ethics board of each participating institution approved the protocol and need for informed consent was according to local rules. [file 13054_2015_922_MOESM1_ESM.pdf]

Participants in three international cohort studies on mechanical ventilation (1998, 2004 and 2010).

Participants in first international study on mechanical ventilation (1998)

Argentina: Coordinators: Carlos Apezteguia, Fernando Palizas.

R. Alasino (Hospital Municipal de Urgencias, Córdoba); R. Bastianelli (Hospital Militar, Villa Revol); J. Berón (Hospital Pablo Soria, San Salvador); C. Bevilacqua (Clínica Modelo de Morón, Morón); M. Cafaro (Hospital Regional Río Gallegos, Río Gallegos); E. Capparelli (Hospital Eva Perón, San Martín); G. Cardonatti (Hospital San Isidro, San Isidro); R. Correa (Hospital Central, Mendoza); A. Díez (Hospital Provincial del Centenario, Rosario); E. Estensoro (Hospital Escuela José de San Martín, La Plata); J. Fara (Policlínico Ferroviario, Rosario); R. Fernández (Hospital Italiano, Guaymallén); G. Fernández Cid (Hospital E. Tomú, Buenos Aires); H. Ferraro (Corporación Médica de San Martín, San Martín); A. Galaverna (Hospital Zonal Bariloche, Bariloche); C. Galleti (Sanatorio Allende, Córdoba); G. García (Hospital Clemente Álvarez, Rosario); G. Gelardi (Hospital Privado del Sur, Bahía Blanca); S. Giannasi (Hospital Italiano, Buenos Aires); R. Guidi (Hospital Italiano Garibaldi, Rosario); L. Huespe Gardel (Hospital Escuela José F. de San Martín, Corrientes); C. Irrazábal (Hospital de Clínicas José de San Martín, Buenos Aires); O. López (Sanatorio Santa Isabel, Buenos Aires); G. Menga (Hospital María Ferrer, Buenos Aires); O. Otero (Centro Oncológico de Excelencia, Gonnet); F. Pálizas (Clínica Bazterrica, Buenos Aires); P. Pardo (Sanatorio de la Trinidad, Buenos Aires); C. Plaza (Sanatorio Julio Méndez, Buenos Aires); G. Raimondi (FLENI, Buenos Aires); A. Raimondi (Sanatorio Mater Dei, Buenos Aires); E. Romero (Hospital Privado Centro Médico, Córdoba); L. de Rosa (Sanatorio Quintar, San Salvador); C. Sáez (Sanatorio Británico, Rosario); A. Sarsino (Hospital Juan A. Fernández, Buenos Aires); P. Schoon (Hospital Prof. Luis Güemes, Haedo); C. Sola (Hospital José Penna, Bahía Blanca); C. Stöltzing (Hospital Guillermo Rawson, San Juan); J. Taccone (Instituto Alfredo Lanari, Buenos Aires); C. Tolosa (Hospital Córdoba, Córdoba); M. Torreno (Sanatorio Modelo Quilmes, Quilmes); E. Turchetto (Hospital Privado de la Comunidad, Mar de Plata); R. Valenti (CEMIC, Buenos Aires); R. Vargas (Policlínico Neuquen, Neuquen); L. Vasta (Sanatorio San Patricio, Buenos Aires); L. Vázquez (Hospital Español, Godoy Cruz); Vetere (Hospital Israelita Ezrah, Buenos Aires); F. Villarejo (Hospital Prof. Alejandro Posadas, Haedo); N. Wainsztein (Hospital Privado Fundación Favaloro, Buenos Aires); O. Yunk (Hospital Español, Buenos Aires); G. Zabert (Clínica Pasteur, Neuquen).

Bolivia: Coordinator: Fredi Sandi

L. Moya (Hospital Juan XXIII, La Paz); E. Salazar (Instituto gastroenterológico boliviano japonés, Santa Cruz); J.C. Zapata (Hospital Obrero No.1, La Paz).

Brazil: Coordinator: Cide M. David.

S.M. Ajeje Lobo (Hosp. de Base de São José do Rio Preto, São José do Rio Preto); A.B. de Almeida (Hospital das Clínicas da Univers. Federal, Uberlândia); M.A. Braga (Hospital Biocor, Belo Horizonte); I. Buselato Chen (Hospital Nossa Senhora das

Graças, Curitiba); M. Chaves Craveiro de Melo (Hospital São Lucas, Belo Horizonte); R.N. Darwich (Hospital Prontocor, Belo Horizonte); C.M. David (Hospital Clementino Fraga Filho, Rio de Janeiro); R. Goldstein Alheira Rocha. (Hospital Samaritano, São Paulo); R. de Macedo Bosco (Hospital Madre Teresa, Belo Horizonte); J.M. Nogueira (Hospital Universitario São José. Belo Horizonte); E. Oliveira (Hospital Vera Cruz, Belo Horizonte); S.F. Pinto (Casa de Saúde São José, Campo Grande); S.F. Pinto (Santa Casa de Campo Grande, Campo Grande); S.F. Pinto (Univ. Fed. Mato Grosso do Sul, Campo Grande); J.L. da Rocha Paranhos (Santa Casa de Misericordia, São João del Rei); L.R. de Siqueira Musolino (Irmandade da Santa Casa de Misericordia, São Paulo).

Canada: Coordinator: Thomas E.Stewart.

R. Fowler (Wellesley-Central Hospital, Toronto). J. Granton (Toronto Hospital General Division, Toronto); J. Granton (Toronto Hospital Western Division, Toronto); R. Hodder (Ottawa Civic Hospital, Ottawa); B. Kashin (Peel Memorial Hospital, Brampton-Ontario); S. Lapinsky (Mount Sinai Hospital, Toronto); D. Mazer (St Michael's Hospital, Toronto); R. McLean (Sunnybrook Health Sciences Centre, Toronto); T. Rogovein (St Joseph's Health Centre, Toronto).

Chile: Coordinator: Luis Soto.

G. Bugedo (Hospital Pontificia Universidad Católica, Santiago); P. Hernández (Instituto Nacional del Tórax, Santiago); C. Ortega (Hospital Regional Concepción, Concepción); L. Soto (Hospital de Coquimbo, Coquimbo); L. Schölz (Hospital de Osorno, Osorno).

Colombia: Coordinator: Marco González.

H. Atehortua (Clínica Sta. María. Centro Cardiovascular, Medellín); C. Cadavid (Hospital Pablo Tobón Uribe, Medellín); D. Camargo (Hospital Universitario, Barranquilla); C. Dueñas (Hospital Universitario, Cartagena); A. Guerra (Hospital General, Medellín); M. Granados (Fundación Valle de Lilly, Cali); R. Panesso (Clínica Las Américas, Medellín); M.A. Perafán (Clínica Shaio, Bogotá).

Ecuador: Coordinator: Jean Raad.

B. Guevara (Hospital Carlos Andrade, Quito); J. Molina & J. Raad (Hospital Militar, Quito)

England: Coordinator: Peter Nightingale.

O. Akinpelu (Chorley & District Hospital, Chorley); D. Bardgett (Macclesfield District General Hospital, Macclesfield); A. Batchelor (Royal Victoria Infirmary, Newcastle upon Tyne); R. Beale (Guy's Hospital, London); K. Burchett (Queen Elizabeth Hospital, King's Lynn); N. Coleman (North Staffordshire Royal Infirmary, Stoke on Trent); A. Conn (Wansbeck General Hospital,

Ashington); D. Edbrooke (Royal Hallamshire Hospital, Sheffield); N. Fergusson (Countess of Chester Hospital, Chester); I. Grant (Rotherham District Hospital, Rotherham); K. Gunning (Addenbrooke's Hospital, Cambridge); J. Harper (Royal Liverpool University Hospital, Liverpool); D. Higgins (Southend Hospital, Westcliffe-on-Sea); D. Jayson (Southport & Formby General Hospital, Southport); R. Loveland (Wexham Park Hospital, Slough); L. Lynch (Birmingham Heartlands Hospital, Birmingham); I. Macartney (North Manchester General Hospital, Manchester); E. Major (Morriston Hospital, Swansea); S. Mousdale (Blackbum Royal Infimary, Blackburn); N. Soni (Chelsea and Westminster Hospital, London); D. Watson (Walsgrave Hospital, Walsgrave).

France: Coordinator: Laurent Brochard.

P. Andrivet (Centre Médico-Chirurgical de Bligny, Bris-sous-Forges); D. Annane (Hôpital Raymond Poincaré, Garches); C. Arich (CHU de Nimes, Nimes); F. Baud (Hôpital Lariboisière, Paris); F. Bellenfant (Hôpital Cochin, Paris); R. Boiteau (Hôpital Louise Michel, Evry); F. Brivet (Hôpital A. Béclère, Clamart); M. Canonne (C.H.G. Les Feugrais, Elbeuf); J.P. Cardinaud (Hôpital Pellegrin-Tripode, Bourdeaux); E. Clémenti (Centre Hosp. Dept, La Roche/Yon); P. Charbonneau (C.H.U. Côte de Nacre, Caen); J. Chastre (Hôpital Bichat, Paris); R. Chauveau (C.H. André Grégoire, Montreuil-Ss-Bois); C. Chopin (CHRU - Hôpital B, Lille); J.M. Descamps (Centre Hospitalier de Niort, Niort); D. Dreyfuss (Hôpital Louis Mourier, Colombes); J.P. Faller (C. Hosp. de Belfort, Belfort); F. Fraisse (Hôpital Delafontaine, Saint-Denis); C. Girault (Hôpital Charles Nicolle, Rouen); C. Guérin (Hôpital Croix Rousse, Lyon); E. Guerot (Hôpital Boucicaut, Paris); F. Hilpert (Hôpital Ballanger, Aulnay-sous-Bois); L. Holzapfel (Centre Hospitalier, Bourg-en-Bresse); F. Jardin (Hôpital Ambroise Paré, Boulogne Vignancourt); O. Jonquet (Hôpital Gui de Chauliac, Montpellier); E. L'Her (CHU de la Cavale Blanche, Brest); Y. Lefort (Hôpital Henri Mondor, Creteil); O. Leroy (Centre Hospitalier, Tourcoing); Y. Le Tulzo (CHU Pontchaillon, Rennes); Ch. Mayaud (Hôpital Tenon, Paris); H. Mentec (Hôpital Victor Dupouy, Argenteuil); A. Mercat Hôpital Bicétre, Kremlin-Bicetre); B. Misset (Hôpital Saint-Joseph, Paris); P. Moine (Hôpital Bicêtre, Bicetre); G. Nitemberg (IGR, Villejuif); L. Papazian (Hôpital Sainte Marguerite, Marseille); A. Rabbat (Hôpital Hôtel-Dieu, Paris); T. Similowski (Hôpital Pitié Salpétrière, Paris); L. Soufir (Hôpital Saint-Louis, Paris); D. Tardy (Hôpital Saint-Camille, Bry-sur-Marne); F. Thaler (CM Chirurgical Foch, Suresnes); B. Vallet (Centre Hospitalier Univ., Lille); D. Villers (C.H.U. Nantes, Nantes); M. Wysocki (Institut Mutualiste Montsouris, Paris); J.F. Zazzo (Hôpital A. Béclère, Clamart).

Greece: Coordinator: D.Matamis.

D. Georgopoulus (Heraklion University Hospital, Heraklion); M. Gianakou (Ahepa University Hospital, Thessaloniki); D. Lagonidis (Papanikolaou Hospital, Thessaloniki); G. Nakos (Ioanina University Hospital, Ioanina); K. Stavrakaki (Evangelismos Hospital, Athens); G. Thomopoulus (Laikon Hospital, Athens).

Ireland: Coordinator: Gerald Fitzpatrick.

M. Donnelly (Adelaide and Meath Hospital, Dublin); J. Moriarty (St. James Hospital, Dublin); B. O'Sullivan (Waterford Regional Hospital, Waterford); G. Shorten (Cork University Hospital, Cork).

Italy: Coordinator: P.Pelosi.

P. Cositi (Pol. Umberto I, Roma); G. Iapichino (Hospital S. Paolo, Milano); P. Pelosi (Policlínico, Milano); A. Pesenti (Dsp. S. Gerardo, Monza).

Mexico: Coordinator: José Elizalde.

F. Aguilera Almazán (Hospital General Regional Nº 1, Chihuahua); M. Benitez Cortazar (Hospital Universitario de Puebla, Puebla); R. Carrillo Speare (Hospital PEMEX Sur, México DF); R. Castaño (Hospital de Cardiología del CMN, México DF); R. Corral (Hospital Excel. Tijuana, Baja California); D.M. D'Ector Lira (Hospital Metropolitano, México DF); G. Díaz Polanco (Hospital de Traumatología Magdalena de las Salinas, México DF); J.J. Elizalde (Hospital ABC, México DF); R. Envila Fisher (Hospital Morelos, Chihuahua); R. Envila Fisher (Hospital Clínica del Parque, Chihuahua); G. Franco G. (Hospital General de México, México DF); P. García Balbuena (Hospital General "Fernando Quiroz", México DF); O. Gayoso Cruz (Hospital Regional "Adolfo López Mateos", México DF); L. Green (Instituto Nacional de Cancerología, México DF); J.O. Herrera Hoyos (Centro Médico Las Américas, Mérida); J. Hinojosa (Hospital Angel Leaño, Guadalajara); J. Huerta (Clínica Londres, México DF); V.A. Juárez (Hospital Santelena, México DF); M. Loera (Hospital General Durango, Durango); C. López Alzate (Clínica del Mar, Mazatlán); E. López Mora (Instituto Nacional de Cardiología, México DF); S. Martínez Cano (Hospital Hidalgo Aguascalientes, Aguascalientes); R. Mendez Reyes (Hospital Regional 1º de Octubre, México DF); M. Mendoza (Hospital General de la Villa, México DF); O. Narváez Porras (Instituto Nacional de Enfermedades Respiratorias, México DF); E. Ortiz (Hospital General Irapuata, Guanajuato); A. Padua (Hospital General Torreón, Coahuila); M. Poblano (Hospital Juárez, México DF); V. Pureco Reyes (Hospital Regional "20 de Noviembre", México DF); W. Querevalum (Hospital Central Cruz Mexicana, México DF); A. Quesada (Hospital Ntra. Sra. de la Salud, San Luis Potosí); A. Ramírez Rivera (Hospital de Enfermedades Cardiovasculares y del Tórax. IMSS, Monterrey); A. Tamariz (Hospital Clínica del Centro, Chihuahua); A. Tamariz (Hospital Central Universitario, Chihuahua); A. Vargas (Hospital General de Pachuca, Pachuca); C. Vázquez (Hospital General Celaya, Guanajuato).

Peru: Coordinator: A.M. Montañez.

M. Contardo (Edgardo Rebagliati Martins - UCI 7ºB, Lima); E. Durand (Guillermo Almenara Irigoyen–IPPS, Lima); M. Manrique (Hospital "Jose Casimiro Ulloa", Lima); J.C. Meza (Centro Médico Naval, Lima); J. Muñoz (Edgardo Rebagliati Martins - UCI 2ºC, Lima); J. Pacheco (Hospital del Apoyo "María Auxiliadora", Lima); C. Salcedo (Hosp. Nacional "Daniel Alcides Carrión", Lima); J. Silva (Hospital Central FAP, Lima); C. Torres (Hospital Nacional "Arzobispo Loayza", Lima).

Portugal: Coordinator: Jose Pimentel.

P. Amaro (Centro Hospitalario de Gaia, Gaia); F. Faria (Instituto Portugués de Oncología, Porto); P. Freitas (Hospital Fernando da Fonseca, Amadora-Sintra); P. Martins (Hospital Universidade, Coimbra); E. Sabino (Hospital García de Orta, Almada); J. Salcher (Hospital de San José. UUM, Lisboa); E. Silva (Hospital Senhora do Desterro, Lisboa).

Spain: Coordinators: Andrés Esteban, Fernando Frutos-Vivar.

J.M.Allegre (Hospital Nuestra Señora del Rosell, Cartagena), S.Alonso (Hospital Joan XXIII, Tarragona), A.Alvarez Ruiz (Hospital General Rio Carrión, Palencia), B.Alvarez Sánchez (Hospital General, Alicante), M.T. Antuna (Hospital de Cabueñes, Gijón), J.M. Añón (Hospital Virgen de la Luz, Cuenca), P.Arribas (Hospital 12 de Octubre, Madrid), A.Ayensa (Hospital Virgen de la Salud, Toledo), A.Azcárate (Hospital Nuestra Señora de Aranzazu, Donostia), J. Blanco (Hospital del Río Hortega, Valladolid), G.M. Besso (Hospital Carlos Haya, Málaga), L. Cabré (Hospital de Barcelona, Barcelona), F. Carrizosa (Hospital General, Jérez de la Frontera), J.Castañeda (Hospital Clínico, Valladolid), R.de Celis (Hospital de Galdakao, Galdakao), J.A. Conesa (Hospital Clínico Universitario San Carlos, Madrid), J.Diarte (Complejo Hospitalario, Ciudad Real), A.Díaz Lamas (Complejo Hospitalario Cristal Piñor, Orense), R. Fernández (Consorci Hospitalari del Parc Taulí, Sabadell), M.Ferrer (Hospital Clinic i Provincial, Barcelona), D.Fontaneda (Hospital Virgen Blanca, León), P.Galdós (Hospital General, Móstoles), A.García Jiménez (Hospital Arquitecto Marcide, El Ferrol), J. García Pardo (Hospital Juan Canalejo, La Coruña), J. Gener (Hospital Germans Trias i Pujol, Badalona), J.A. Gómez Rubí (Hospital Virgen de la Arrixaca, Murcia), G.González Díaz (Hospital Morales Meseguer, Murcia), S.González Prado (Hospital Josep Trueta, Girona), C.Homs (Hospital General San Jorge, Huesca), J. Ibañez (Hospital Son Dureta, Palma de Mallorca), F. Jara (Hospital Mutua, Terrassa), M.León (Hospital Arnau de Vilanova, Lleida), A.Lloria (Complejo Hospitalario Rebullón, Pontevedra), J. López Díaz (Hospital La Paz, Madrid), MªR. Lorenzo (Complejo Hospitalario Materno-Infantil, Las Palmas de Gran Canaria), S. Macías (Hospital General, Segovia), J.A. Maldonado (Hospital de la Serranía, Ronda), J. Maynar (Hospital Santiago Apostol, Vitoria), A.Moreno (Complejo Hospitalario de San Millán-San Pedro, Logroño), A.Mota (Hospital General Universitario, Elche), T.Mut (Hospital General, Castellón), M.Nolla (Hospital General de Cataluña, Sant Cugat del Vallés), F. Ortega (Hospital Universitario de Valme, Sevilla), R. de Pablo (Hospital Príncipe de Asturias, Alcalá de Henares), E.Palazón (Hospital General Universitario, Murcia), V.Parra (Hospital de Sagunto, Sagunto), A.Peral (Hospital Gregorio Marañón, Madrid), J.C. Portela (Complejo Hospitalario Xeral-Calde, Lugo), A.Ramírez (Hospital Nuestra Señora de Sonsoles, Avila), J.A. Ramos (Hospital de Poniente, El Ejido), P. Revuelta (Hospital Universitario de Canarias, La Laguna), M. Rey (Complejo Hospitalario, Santiago de Compostela), J.J. Rodrigo (Hospital Nuestra Señora del Pino, Las Palmas de Gran Canaria), J.C. Rodríguez Borregan (Hospital Marqués de Valdecilla, Santander), J.A. Rodríguez Sarria (Hospital General, Elda), A.Rubio (Hospital Ramón y Cajal, Madrid), S.Ruiz Navarro (Hospital General Ciudad de Jaen, Jaen), V.Sagredo (Hospital Virgen de la Vega, Salamanca), P. Saura (Centre Hospitalari, Manresa), M.J. Serralta (Hospital Universitario de San Juan, Alicante), J.F. Solsona (Hospital del Mar, Barcelona), F. Suárez Sipmann (Fundación Jiménez Díaz, Madrid), F. Taboada (Hospital General de Asturias, Oviedo), S.Temprano (Hospital Severo Ochoa, Leganés), J.P. Tirapu (Hospital de Navarra, Pamplona), MªV. de la Torre

(Hospital Universitario Virgen de la Victoria, Málaga), P.Ugarte (Hospital Marqués de Valdecilla, Santander), M. Valledor (Hospital de San Agustín, Avilés), I.Vallverdú (Hospital de la Santa Creu i Sant Pau, Barcelona), C.Vaquerizo (Hospital 12 de Octubre, Madrid), A.Viñuales (Hospital Lluis Alcanyis, Xátiva).

Tunisia: Coordinator: Fekri Abroug.

A. Bchiz (Hospital F. Bached, Sousse); J. Ben Khelil (Hospital A. Mami, Ariana); S. Bern Lakhal (Hospital Rabta, Tunis); B. Bouhaja (Hospital Mongi Slim, La Marsa); H. Chelly (Hospital Fattouma Bourguiba, Sfax); S. El Atrous (Hospital Fattouma Bourguiba, Monastir); S. Ghedira (Hospital Charles Nicolle, Tunis); H. Thabet (CAMUR. Tunis).

Uruguay: Coordinator: Carlos Rodrigo.

H. Bagnulo (Maciel, Montevideo); C. Rodrigo (Asociación Española Primera, Montevideo); M. Rodríguez (Hospital de Paysandú, Montevideo).

Unites States: Coordinator: Antonio Anzueto.

S.M. Aguayo (Atlanta VA Medical Center, Decatur); R. Alagar (Allegheny General Hospital, Pittsburgh); R.K. Albert (Denver Health Medical Center, Denver); T.K. Aldrich (Montefiore Hospital & Medical Center, Bronx); K. Amoosa (Medical College of Wisconsin, Milwaukee); N. Anandarao (New York Methodist Hospital, Brooklyn); D.C. Angus (University of Pittsburg, Pittsburgh); A.C. Arroliga (Cleveland Clinic Foundation, Cleveland); M.F. Azrieli (Jacobu Medical Center, Bronx); R.A. Balk (Medical Center – 203 Jelke, Chicago); P.W. Bates (Maine Medical Center, Porthland); J.F. Beamis, Jr. (Lahey Hitchcock Medical Center, Burlington); P.E. Bellamis (Chs Dept of Medicine, Los Angeles); D.J. Bower (Atlanta VA Medical Center, Decatur); J.P. Bradley (William Beaumont Medical Center, El Paso); R.P. Byrd, Jr.(University of East Tennese, Jonesboro); V.J. Cardenas, Jr.(University of Texas Medical Branch, Galveston); L.J. Caruso (University of Florida, Gainesville); B.R. Celli (St. Elizabeths Medical Center, Boston); G. Clermon (University of Pittsburg, Pittsburgh); S.J. Coole (Carl T. Hayden VA Medical Center, Phoenix); T.A. Dillard (Commander MCHJ-MPU, Tacoma); L.E. Efferen (SUNY Health Science Center, Brooklyn); E.W. Ely, Jr.(Vanderbilt Lung Transplant Program Newline, Nashville); P. Factor (Michael Reese Hospital & Medical Center, Chicago); T.M. Fitzpatrick (Walter Reed Army Medical Ctr, Whashington); R. Fowler (Wellesley-Central, Toronto); G.N. Giacooppe, Jr. (MCHJ-MPU, Tacoma); K.K. Guntupalli (Texas Med Ctr - Ben Taub Gen Hospital, Houston); J.B. Hall (University of Chicago, Chicago); M.E. Hanley (Denver Medical Center, Denver); M.T. Haupt (Oregon Health Science University,Portland); G.B. Hayes (St. Elizabeths Medical Center, Boston); D.E. Heiselman (Akron General Medical Center, Akron); F.C. Hiller (University of Arkansas Med Science, Little Rock); J.D. Hinze (The Univesity of Texas Health Science Center at San Antonio, San Antonio); R.D. Hite (Bowman Gray School of Medicine, Winston-Salem); R.C. Hyzy (Henry Ford Hospital, Detroit); A. Jubran (Edward Hines VA Hospital, Hines); C.A. Kaplan (University of Missouri Columbia, Columbia); M.S. Karetzky (Newark Beth Israel Med

Ctr, Newark); S.A.Kurenhy (Truman Medical Center, Kansas); K.V. Leeper, Jr.(Emory University School of Medicine, Atlanta); H. Levy (University of New Mexico, Alburquerque); T. Lo (Loma Linda University, Loma Linda); M.J. Mador (Buffalo VA Medical Center, Búfalo); G.P. Marelich (University of California Davis Med Ctr, Sacramento); M.A. Matthay (University of California, San Francisco); N.R. McIntyre (Duke University Medical Center, Durham); S.A. Metter (Maine Medical Center, Portland); M.S. Niederman (Winthrop Univesity Hospital, Mineola); J.R. Norman (Univesity of Mississippi Medical Center, Jackson); D.R. Oullette (Brooke Army Medical Center, Fort Sam Houston); P. Parsons (Denver Medical Cente, rDenver); R.G. Patel (VA Medical Center, Jackson); R.C. Perkins,II (University of Texas Health Center at Tyler, Tyler); M.E. Petrini (University of Miississippi Medical Center, Jackson); M.R. Pinsky (University of Pittsburg, Pittsburgh); A. Pohlman (Edward Hines VA Hospital, Hines); K.W.Presberg (Medical College of Wisconsin, Milwaukee); M.P. Rocha (Carl T. Hayden VA Medical Center, Phoenix); W. Rodríguez Cintron (San Juan VA Medical Center, San Juan); M.J. Rosen(Beth Israel Medical Center, New York); T.M. Roy (James Quillen College of Medicine, Mountain Home); G. Rubenfeld (Harborview Medical Center, Seattle); M.J. Rumbak (University Florida, Tampa); S.J. Ruoss (Stanford University Medical Center, Stanford); G.A. Schmidt (University of Chicago, Chicago); R.F. Schneider (Beth Israel Medical Center, New York); C.N. Sessler (Medical College of Virginia, Richmond); C.S. Shim (Jacobi Medical Center, Bronx); L. Smith (Rush-Presbyterian-St Lukes Medical Center, Chicago); C. Strange (MUSC 96 Jonathan Lucas St, Charleston); J.I. Sznajder (Michel Reese Hospital & Medical Center, Chicago); S. Tessler (Maimonides Medical Center, Brooklyn); V.Whyte (Loma Linda University, Loma Linda); L. Wilkelmeyer (Loma Linda University Medical Center MC 1521, Loma Linda); R.G. Wundering (501 Crews Wing, Memphis); M.H. Zaman (The Brookdale Hosp Med Ctr, Brooklyn); L.H. Zimmerman (San Francisco VA Medical Center, San Francisco).

Venezuela: Coordinator: Gabriel D´Empaire.

J. España (Hospital Universitario, Caracas); F. Pérez (Hospital de Clínicas, Caracas); R. Zerpa (Hospital Militar, Caracas).

Participants in the second international study on mechanical ventilation (2004).

Argentina: Coordinators: Carlos Apezteguia and Pablo Desmery

A. Sarasino and D. Ceraso (Hospital Dr. Juan A. Fernández, Buenos Aires), D. Pezzola and F.Villarejo (Hospital Prof. A. Posadas, El Palomar), C. Cozzani and M. Torres Boden (Hospital Dr. C. Argerich, Buenos Aires), C. Santos and E. Capparelli (Hospital Eva Perón, San Martín), M. Tavella and C. Irrazábal (Hospital de Clínicas José de San Martín, Buenos Aires), L. Cardonnet and A. Diez (Hospital Provincial del Centenario, Rosario), A. Giannelli and L. Vargas (Policlínico de Neuquén), M. Bustamante (Hospital Héroes de Malvinas, Merlo), E. Turchetto (Hospital Privado de la Comunidad, Mar del Plata), J. Teves and O. Elefante (Hospital Oscar Alende, Mar del Plata), C. Sola and J. Mele (Hospital Dr. José Penna, Bahía Blanca), V. Sciuto and P. Grana (Hospital Provincial de Neuquén), G. Jannello and R. Valentini (CEMIC, Buenos Aires), S. Ilutovich (Sanatorio Mitre, Buenos Aires), L. Huespe Gardel (Hospital Escuela José F. de San Martín, Corrientes), J. Scapellato and E. Orsini (Hospital F. Santojanni,

Buenos Aires), G. Agüero and Á. Sánchez (Policlínico Regional J. Perón, Mercedes), R. Fernández and L. Villalobos Castañeda (Hospital Italiano, Buenos Aires), F. González and E.Estenssoro (Hospital General San Martín, La Plata), S. Lasdica (Hospital Privado del Sur, Bahía Blanca), A. Gómez and J. Scapellato (Clínica de la Esperanza, Buenos Aires), P. Pratesi (Hospital Universitario Austral, Pilar), M. Blasco and F. Villarejo (Clínica Olivos, Olivos), G. Olarte and C. Bevilacqua (Clínica Modelo de Morón / Hospital San Juan de Dios, R.Mejía), M. Quinteros (Sanatorio San Lucas, San Isidro) , P. Ripoll (Clínica La Sagrada Familia, Buenos Aires), S. Filippus (Clínica del Valle, Comodoro Rivadavia), F. Guzman Díaz and M.Deheza (Hospital B. Rivadavia, Buenos Aires), E. García and J. Arrieta (Hospital Regional de Comodoro Rivadavia), P. Pardo and J. Neira (Sanatorio de la Trinidad de Palermo, Buenos Aires), J. Núñez and F. Pálizas (Clínica Bazterrica, Buenos Aires), A. Ciccolini and G.Murias (Sanatorio Santa Isabel, Buenos Aires), W. Vázquez and M. Grilli (Hospital Español de Mendoza, Godoy Cruz), F. Chertcoff and E. Soloaga (Hospital Británico, Buenos Aires), D. Vargas and J. Berón (Hospital Pablo Soria, San Salvador de Jujuy), A. Maceira and P. Schoon (Hospital Prof. Luis Güemes, Haedo), D. Pina (Sanatorio Franchín, Buenos Aires), E. Sobrino and A.Raimondi (Sanatorio Mater Dei, Buenos Aires), E. De Vito (IIM Alfredo Lanari, Buenos Aires).

Belgium: M. Malbrain (Ziekenhuis Netwerk, Antwerpen)

Bolivia: Coordinator: Freddy Sandi

A. Lavandez and C. Alfaro (Complejo Hospitalario Viedma, La Paz), J. Guerra (Instituto gastroenterológico boliviano japonés, Santa Cruz).

Canada: Coordinators: Niall D.Ferguson and Maureen O. Meade

J.T. Granton (Toronto General Hospital), S. E. Lapinsky (Mount Sinai, Toronto), J. Meyer (St. Joseph´s Hospital, Toronto), D.C. Scales (St. Michael´s Hospital, Toronto), R.A. Fowler (Sunnybrook Health Sciences Centre, Toronto), B. Kashin (William Osler Health Centre, Brampton, Ontario), D. J. Cook (St. Joseph's Healthcare, Hamilton)

Chile: Coordinator: Vinko Tomicic

L.Soto (Instituto Nacional del Tórax, Santiago), C. Romero (Hospital Clínico Pontificia Universidad Católica, Santiago), M.Teresa Caballero and L. Chiang (Hospital naval almirante NEF), E. Poch (Instituto de Neurocirugía), J. Canteros Gatica (Hospital Curico), H. Ugarte (Hospital de Coquimbo), M. Calvo, C. Vargas and M.Yacsich. (Hospital Regional de Valdivia), E. Tobar (Hospital Clínico de la Universidad de Chile, Santiago), J. G. Urra (Clínica Alemana de Temuco)

Colombia: Coordinator Marco A. González

A. Guerra (Hospital General de Medellín and Clínica SOMA, Medellín), C. Cadavid (Hospital Pablo Tobón Uribe, Medellín), R. Panesso (Clínica Las Américas, Medellín), M.Granados (Clínica Valle del Lilli, Cali), C. Dueñas (Hospital Bocagrande,

Cartagena), F. Molina (Clínica Bolivariana, Medellín), R. Camargo (Clínica General del Norte de Barranquilla), G. Ortiz (Hospital de Santa Clara, Bogotá), M. Gómez (Hospital de San José).

England: Coordinator: Peter Nightingale

J. Hunter (Macclesfield District General Hospital, Macclesfield), J. Hunter (Rotherdam District General Hospital, Rotherdam), S. Mousdale (Blackburn Royal Infimary, Blackburn), J. Harper (Royal Liverpool University Hospital, Liverpool), A. Conn (Wansbeck General Hospital, Ashington), D. Higgins (Southend Hospital, Westcliffe-on-Sea), D.Jayson (Southport & Formby District General Hospital, Southport), D. Hawkins (North Staffordshire Hospital, Stoke on Trent).

Ecuador: Coordinator: Manuel Jibaja

G. Paredes and E. Bazantes (Hospital Enrique Garcés, Quito), P. Jiménez (Hospital Carlos Andrade Martín, Quito), J. Vergara and L. González (Hospital Luis Vernaza Valdez, Guayaquil)

France: Coordinators Laurent Brochard and Arnaud Thille

L. Mallet (Centre Hospitalier D'Auch), P. Andrivet (Centre Médico-Chirurgical de Bligny, Bris-sous-Forges), O. Peyrouset (Hôpital Ambroise Paré, Boulogne Billancourt), I. Mohammedi (Hôpital Edouard Herriot, Lyon), E. Guerot (Hôpital Européen Georges Pompidou , Paris), N. Deye (Hôpital Lariboisière, Paris), S. Monsel and F. Bouvet (Hôpital Pitié Salpétrière, Paris), M. Darmon (Hôpital Saint Louis, Paris), M. Fartoukh and A. Harb (Hôpital Tenon, Paris), N. Anguel (Hôpital de Bicêtre, Kremlin-Bicêtre).

Germany: Coordinator: Konstantinos Raymondos

A. Nowak, T. Pahlitzsch and K. F. Rothe (Krankenhaus Dresden-Friedrichstadt), M. Ragaller and T. Koch (Universitaetsklinikum Carl Gustav Carus Dresden), G. Sterzel (Kreiskrankenhaus Loebau, Ebersbach), R. Wittich (Carl-Thiem-Klinikum Cottbus gGmbH), K. Rudolph and J. Raumanns (St. Elisabeth gGmbH Leipzig), U. Grueneisen and F. Stupacher (Bundeswehrkrankenhaus Leipzig), H. Bromber, G. Leonhardt and J. Soukup (Universitaetsklinikum der Martin-Luther-Universitaet Halle-Wittenberg), C. Wuttke (Krankenhaus St. Elisabeth und St. Barbara Halle, Saale), M. Holler (Staedtisches Krankenhaus Martha-Maria Halle-Doelau gGmbH), J. Haberkorn (Georgius-Agricola-Klinikum Zeitz), P. Jehle (Paul-Gerhard-Stiftung, Lutherstadt Wittenberg), B. Albrecht (Zeisigwaldkliniken Bethanien Chemnitz), D.M. Klut (Kreiskrankenhaus Rochlitz), H. J. Hartung (Vivantes Krankenhaus am Urban, Berlin-Kreuzberg), H. Gerlach (Vivantes-Klinikum Neukoelln, Berlin), T. Henneberg, S. Weber-Carstens, K. Haid, and C. Melzer-Gartzke, M. Oppert (Charité Universitaetsklinikum, Campus Virchow, Berlin), M. Reffenberg (Lungenklinik Heckeshorn, Berlin), Ch. Werel and A. Kopietz (Klinikum Barnim GmbH, Werner Forßmann Krankenhaus, Eberswalde), T. Nippraschk and D. Hoffmeister (Ruppiner Klinikum GmbH, Neuruppin), M. Schneider (Dietrich-Bonhoeffer-

Klinikum-Neubrandenburg), D. A. Vagts and G. Noeldge-Schomburg (Medizinische Fakultaet der Universitaet Rostock), G. Savinski and T. Kloess (Allgemeines Krankenhaus Harburg, Hamburg), C. Frenkel, D. Yakisan, H. Schroeder and C. Daniels (Staedtisches Klinikum Lueneburg), B. Sedemund-Adib (Universitaetsklinikum Schleswig Holstein - Campus Luebeck), S. Krueper (Klinikum Hannover Nordstadt), J. Ahrens, U. Molitoris and K. Johanning (Medizinische Hochschule Hannover), D. Korth and W. Seitz (Kreiskrankenhaus Hameln), J. Kleideiter and P. Palomino (Staedtische Kliniken Bielefeld gGmbH), A. Lunkeit and J. Schlechtweg (Klinikum Bad Salzungen gGmbH), M. Quintel (Universitaetsklinikum der Georg-August-Universitaet Goettingen), E. Schild and C.P. Criée (Evangelisches Krankenhaus Goettingen-Weende e.V., Bovenden-Lenglern), M. Bund (Albert-Schweitzer-Krankenhaus Northeim), M. Hundt, U. Schulze and J. Kolle (Kreiskrankenhaus Charlottenstift, Stadtoldendorf), J. Offensand, S. Youssef, and J.P. Juvana (Klinikum Salzgitter GMBH), W. Seyde (Staedtisches Klinikum Wolfenbuettel), T. Luecke and A. Gruener (Universitaetsklinikum Mannheim), E. Calzia (Universitaetsklinikum fur Anasthesiologie, Ulm), J. Heine, M. Borth, U. von Leitner and M. Hoffmann (Dr. Herbert-Nieper-Krankenhaus-Goslar), W. Brandt (Universitaetsklinikum Magdeburg), A. Keller and S. Scieszka (Krankenhaus Neuwerk, Moenchengladbach), E. Schroeder and F. L. Deres (Kreiskrankenhaus Dormagen), M. Burrichter, T. Bernhardt and W. Wilhelm (St.-Marien-Hospital, Luenen), M. Beiderlinden (Universitaetklinikum Essen), H. Steiniger and V. Weißkopf (Ruhrlandklinik, Essen), H. Militzer (Evangelisches und Johanniter Klinikum, Dinslaken), K. Eicker and F. Hinder (Universitaetsklinikum Muenster), C. Weilbach and M. Raab (St. Josefs-Stift Cloppenburg), F. Ragalmuto (Kliniken der Stadt Koeln Krankenhaus Holweide), T. Moellhoff and K. Tsompanidis (Katholische Stiftung Marienhospital Aachen), D. Henzler and R. Kuhlen (Universitaetsklinikum Aachen), H. Wrigge, C. Putensen and F. L. Dumoulin (Universitaetsklinikum Bonn), M. Foedisch and J. Busch (Evangelisches Waldkrankenhaus Bad Godesberg gGmbH, Bonn), W. Theelen (St. Johannes-Krankenhaus Troisdorf), A. Deller (Krankenhaus der Barmherzigen Brueder, Trier), W. Baier (St. Nikolaus-Stiftshospital GmbH, Andernach), B. Eller (Staedt. Hellmig-Krankenhaus, Kamen), K. Schwarke (Evang. Krankenhaus Schwerte GmbH), J. Büttner (Evangelisches Krankenhaus Elisabethenstift gGmbH, Darmstadt), K. P. Wresch and K. Steidel (St.-Vincentius-Krankenhaus Speyer), J. F. Meyer (Universitaetsklinikum der Ruprecht-Karls-Universitaet Heidelberg), M. Layer (Thoraxklinik Heidelberg gGmbH), G. Meinhardt (Robert-Bosch-Krankenhaus, Stuttgart), J. Fritschi and P. Zaar (Ermstalklinik Staedtisches Krankenhaus Sindelfingen), H. P. Stegbauer (Kreiskrankenhaus Leonberg), V. Tumbass and S. Hahn (Ermstalklinik Bad Urach), H. Mende, M. Fischer, J. Martin and A. Assmann (Klinik am Eichert Goeppingen), V. Schoeffel, K. van Deyk and S. Seyboth (Stadtklinik Baden-Baden), H. Kerger and J. Ernst (Evangelisches Diakoniekrankenhaus, Freiburg), H. F. Ginz (Kreiskrankenhaus Loerrach), F. Brettner (Krankenhaus der Barmherzigen Brueder, Muenchen), O. Karg (ASKLEPIOS Fachkliniken Muenchen-Gauting), M. Glaser and T. P. Zucker (Klinikum Traunstein), J. Jahn and A. Schneider (Fachkliniken Wangen), M. Burkert (Bundeswehrkrankenhaus Ulm), H. Kuenzig and T. Bein (Klinikum der Universitaet Regensburg), A. Speicher (Krankenhaus der Barmherzigen Brueder, Regensburg), J. Brederlau, E. Kaufmann, F. Schuster and C. Soellmann

(Universitaetsklinik Wuerzburg), S. Frenzel and L. Pfeiffer (Unstrut-Hainich Kreiskrankenhaus Muehlhausen), S. Weber-Carstens, K. Haid, C. Melzer-Gartzke, C. von Heymann and B. Temmesfeld (Charité Universitaetsklinikum, Campus Mitte, Berlin).

Greece: Coordinator: Dimitrios Matamis

H. Mouloudi (Ippokration General Hospital, Athens)

Italy: Coordinator: Paolo Pelosi

A.Pesenti and N. Rossi (Ospedale San Gerardo, Monza), D. Chiumello and L. Gattinoni (Ospedale Maggiore Policlinico, Milano), P. Severgnini (Ospedale di Circolo di Varese), R. Fumagalli and A. Nikiforov (Ospedali Riuniti di Bergamo), S.Grasso (Ospedale di Venere, Bari).

Mexico: Coordinator: José Elizalde

P. Cerda (Centro Médico de las Américas, Mérida), R. Mercado (Hospital Universitario de Monterrey), J.Albe Castañón (Instituto mexicano del seguro social HECMNS XXI, México DF).

Netherlands: Michael Kuiper, P.H.M. Egbers and M. Koopmans (Medical Center Leeuwarden)

Peru: Coordinator: Ana María Montañez

M. Contardo, J. Cerna and R.Roldán (Hospital Edgardo Rebagliati Martins, Lima), J.Zevallos and S.Alcabes (Hospital Guillermo Almenara Irigoyen, La Victoria), C.Salcedo and D.Bruzone (Hospital Nacional Daniel Alcides Carrión, Callao), J.Quiñones (Hospital de Emergencias Grau, Lima), M.Suárez Lazo (Hospital Nacional Hipólito Unanue, El Agustino), A.Cifuentes (Hospital de Emergencias José Casimiro Ulloa, Miraflores), M.Mayorga (Clínica San Pablo, Lima).

Portugal: Coordinator: Rui Moreno

P.Casanova (Hospitais da Universidade de Coimbra), R. Matos and A.L. Jardim (Hospital de Santo António dos Capuchos, UCIP, Lisboa), A. Godinho (Hospital dos SAMS, UCI, Lisboa), P. Póvoa (Hospital São Francisco Xavier, UCIM, Lisboa), P. Coutinho (Centro Hospitalar de Coimbra), L. Reis (Hospital de São José, Unidade de Urgência Médica, Lisboa).

Saudi Arabia: Coordinator: Yaseen Arabi

N.Abouchala (King Faisal Hospital), F. Hameed (King Khalid National Guard Hospital)

Spain: Coordinators: Nicolas Nin and Eva Tejerina

F. Gordo (Fundación Hospital de Alcorcón), R. Fernandez (Complejo Hospitalario Parc Taulí, Sabadell), R. de Pablo (Hospital Universitario Príncipe de Asturias, Alcalá de Henares), J. Ibañez (Hospital Son Dureta, Palma de Mallorca), E. Fernández Mondejar

(Hospital Virgen de las Nieves, Granada), F. del Nogal (Hospital Severo Ochoa, Leganés), F. Taboada (Hospital Central de Asturias, Oviedo), A. García Jiménez (Hospital Arquitecto Marcide, El Ferrol), Ll. Cabré and J. Morillas (Hospital de Barcelona-SCIAS), S. Macias (Hospital General de Segovia), R. de Celis (Hospital de Galdakao), J. M.Añón (Hospital Virgen de la Luz, Cuenca),P. Ugarte (Hospital Marqués de Valdecilla, Santander), T. Mut (Hospital de la Plana, Vila-Real), J. Diarte (Complejo Hospitalario de Ciudad Real), V. Sagredo (Hospital Clínico de Salamanca), M. Valledor (Hospital San Agustín, Avilés), G.González and L. Rodríguez (Hospital Morales Meseguer, Murcia), V.Parra and E. Gómez (Hospital de Sagunto), F. Jara (Hospital Mutua de Terrassa), J.M. Quiroga (Hospital de Cabueñes, Gijón), L. Arnaiz (Hospital Clínico Universitario de San Carlos, Madrid), Á. Ayensa (Hospital Virgen de la Salud, Toledo), F. Suárez Sippman (Fundación Jiménez Díaz), F. Charizosa (Hospital General de Jerez de la Frontera), J. A. Rodríguez Sarría (Hospital de Elda), C. Homs (Hospital San Jorge, Huesca), A. Díaz Lamas (Hospital Cristal Piñor, Ourense), M. León (Hospital Arnau de Vilanova, Lleida), J. Allegue (Hospital Nuestra Señora del Rosell, Cartagena), M.Ruano (Hospital La Fe, Valencia).

Tunisia: Coordinator: Fekri Abroug

M.Besbes, J. Ben Khelil, K. Belkhouja and K. BenRomdhane (Hospital Abderrahmane Mami, Ariana), S. Ben Lakhal, S. Abdellatif and K. Bousselmi (La Rabta Tunis), M.Amamou and H.Thabet (CAMUR), L. Besbes and N. Nciri (Fattouma Bourguiba Monastir), M.Bouaziz, H. Kallel and M. Bahloul (Habib Bourguiba Sfax), S. ElAtrous, S.Merghli and M. Feki Hassen (Tahar Sfar Mahdia).

Turkey: Coordinator: Nahit Cakar

R. Iscimen (Uludag University School of Medicine, Bursa), M. Kyzylkaya (College of medicine, Ataturk University, Erzurum), B. Yelken (Osmangazi University, Eskisemir), I. Kati (Medical Faculty of Yuzuncu Yil University, Van) , T. Guldem (Haydarpasa Numune Teaching and Research Hospital, Istambul), U. Koca (Dokuz Eylun University, Istanbul), M.Cicek (Inonu University of Medical Faculty, Malatya), H. Sungurtekin (Pamukkale University Medical Faculty)

United States: Coordinator: Antonio Anzueto

A.C. Arroliga (Cleveland Clinic, Cleveland), O. Gajic and M. Ali (Mayo Clinic, Rochester), D. Ost, A.Fein, A. Kyprianou, L. Shulman and S. Chang (North Shore University Hospital, New York), J.S. Steingrub, M.A. Tidswell and K. Kozikowski (Baystate Medical Center, Springfield), C.A. Piquette and L.Morrow (Creighton University Medical Center, Nebraska), P.Scheinberg and J.Green (Saint Joseph's Hospital, Atlanta), L.Penogreen and K. Kannady (Georgia State University Kennestone), M. Moss, M. Mealer, and R.D. Restrepo (Grady Hospital Georgia, Atlanta), H.E. Fessler, R. Brower, D. Hager and A. Scully (John Hopkins University Hospital, Baltimore), J. Beamis, D.E. Craven andW. Miner (Lahey Clinic Medical Center, Burlington), S. Blosser, K. Miller, L.Cornman and J.Breidinger (Penn State Hershey Medical Center, Hershey), J.T. Huggins and Ch.Strange (Medical University of South Carolina, Charleston), N.S. Hill and L.Lawler (Tufts-New England Medical Center, Boston), M.

Rembert (Newark Beth Israel Medical Center), H.K. Donnelly, J.D. D'Amico, R.G. Wunderink, N.Queseda and J. Topin (Northwestern Memorial Home Health University, Chicago), G.T. Kinasewitz and G.L. Lee (University of Oklahoma Health Sciences Center, Oklahoma City), J.Walls and V. Zimmer (Presbyterian Healthcare, Charlotte), A.X. Freire (Regional Medical Center, Memphis), C. Steven and L. Caskey (Louisiana State University Health Sciences Center, Shreveport), R. Dhand and L.A. Despins (University Hospital and Clinics MU Healthcare, Columbia), R.Hyzy, R.E. Dechert, C.Haas and D. Fickle (University of Michigan Medical Center), Ch. Burger and L. Gambino (Mayo Clinic, Jacksonville), D. Marks and S. Benslimane (University of Texas Health Science Center, San Antonio), V.J. Cardenas Jr. (University of Texas Medical Branch Galveston), M.J. Wing and P.Krumpe (VA Sierra Nevada Health Care System, Reno), J. Truwit and M. Marshall (University of Virginia Health System, Charlottesville), D.L. Herr (Washington Hospital Center, Washington DC), RD Hite (Wake Forest Baptist Hospital Medical Center, Winston Salem), PJ McShane and KN Olivier (Wilford Hall Medical Center, Texas), KW Presberg (Froedtert & Medical College, Milwaukee).

Uruguay: Coordinator: Javier Hurtado

M. Borde, E.Echavarría, S. Gómez and M. Berón (Hospital Maciel, Montevideo), F. Villalba (Sanatorio Casa de Galicia, Montevideo), I. Porras (Sanatorio CASMU 2, Montevideo), P. Cardinal, C. Surraco and V. Navarrete (Sanatorio CASMU 4, Montevideo), F. Rodríguez and J.C. Bagattini (Hospital Británico, Montevideo), R. Garrido (Hospital Evangélico and Sanatorio IMPASA, Montevideo), S. Infanzón and J. Caraballo (Hospital Militar and CTI-SMI, Montevideo), C. Santos and A. García (Hospital de Clínicas, Montevideo), R. Cal (CTI-SMI, Montevideo), G. Pittini and J. Cabrera (Centro Nacional de Quemados, Montevideo), F. Bazzano and F. Domínguez (Hospital Pasteur, Colonia), P. Alzugaray, D. González and M.Machado (Sanatorio CAMOC, Carmelo), F.Torres (Sanatorio Mautone and Asistencial Medica de Maldonado, Maldonado), S. Mareque, M. Korintan, F.Mora, E. Altieri, E. Gianoni, C. Fregosi, A. Crossi, G. Larrarte (Sanatorio CAAMS, Soriano), O. Pereira (Sanatorio COMTA, Tacuarembó), J. Baraibar (Hospital Regional de Tacuarembó), A. Soler (Sanatorio COMEPA, Paysandú), M. Rodríguez Verde (Hospital Paysandú), M. Díaz (Hospital de Salto and Sanatorio Uruguay, Salto), J. Martínez Ramos (Sanatorio Uruguay, Salto), I. Iturralde, W. González and E. Cubas (Sanatorio CAMDEL, Minas), A. Cataldo (Sanatorio CAMEDUR, Durazno), O. Rocha (Sanatorio GREMEDA, Artigas), A.Deicas (Sanatorio CASMU 2 and Sanatorio CASMU 4)

Venezuela: Coordinator Gabriel D'Empaire

R. Zerpa (Hospital Militar de Caracas), M. Narvez (Hospital Domingo Luciani, Caracas), F.Pérez (Hospital de Clínicas, Caracas), J. España (Hospital Universitario de Caracas).

Participants in the third international study on mechanical ventilation (2010):

Argentina*:* Coordinators: Fernando Ríos and Damian Violi

Marisol Rodríguez-Goñi, Roger Lamoglie & Fernando Villarejo (Hospital Nacional Profesor A. Posadas, Buenos Aires); Norberto Tiribelli & Santiago Ilutovich (Sanatorio de la Trinidad, General Mitre); Matías Brizuela & Mariana Monllau (Hospital Tránsito Cáceres de Allende, Córdoba); Fernando Saldarini & Silvina Borello (Hospital General de Agudos donación Francisco Santojanni, Buenos Aires); Alberto Marino & Norberto Tiribelli (Hospital Churruca-Visca, Buenos Aires); Mauricio Vinzio & Karina Bonasegla (Sanatorio de la Trinidad, San Isidro); Julián Hernández & María Belén Yapur (Sanatorio Nuestra Señora del Rosario, Jujuy); María Eugenia González (Hospital Privado de Comunidad, Mar del Plata); Sebastián E. Mare (Sanatorio Dr. Julio Méndez, Buenos Aires); Judith Sagardía & Marco Bezzi (Hospital General de Agudos P. Piñeiro, Buenos Aires); Cecilia Pereyra & Julian Strati (Hospital Interzonal General de Agudo Profesor Dr .Luis Guemes, Haedo); Daniel Vargas & Claudia Diaz (Hospital Pablo Soria, Jujuy); Pablo Gómez & Marcelo Palavecino (Sanatorio Juncal, Temperley); Graciela Elizabeth & Aguilera García, M. Eugenia (Hospital de San Luis); Luis Pablo Cardonnet & Lisandro Betttini (Hospital Provincial del Centenario, Rosario); Hernán Nuñez & Lucas Vallejo (Hospital General de Agudos Juan A. Fernández, Buenos Aires); Fernando Fernández & Jorge Arroyo (Hospital Central, Mendoza); Daniel Duarte & Gerardo Filippa (Hospital Regional Río Grande, Tierra del Fuego); Cayetano Galetti & Hernan Nunia (Sanatorio Allende, Córdoba); Fernando Lambert & Elisa Estenssoro (Hospital Interzonal de Agudos San Martin, La Plata); Marina Busico & Fernando Villarejo (Clínica Olivos, Vicente López); Javier Horacio Álvarez (Hospital Universitario Austral, Pilar); Alejandro Raimondi & Gustavo Badariotti (Sanatorio Mater Dei, Buenos Aires); Martín Lugaro (Sanatorio Las Lomas, San Isidro); Fernando Lipovestky (Clínica Santa Isabel; Buenos Aires); Alan Javier Zazu & Hugo Capponcelli (Clínica Privada de Especialidades de Villa María); Patricia Vogl & Cristina Orlandi (Hospital Zonal Francisco López Lima, General Roca); Alejandro Gómez & Gustavo Jannello (Sanatorio de los Arcos; Buenos Aires); Alejandro Risso (Sanatorio Otamendi y Miroli, Buenos Aires); Leticia Rapetti & Guillermo Chiappero (Hospital Universitario, Universidad Abierta Interamericana, Buenos Aires); Juan Domingo Fernández (Hospital Regional de Comodoro Rivadavia, Chubut); Rodrigo E. Gómez-Paz (Hospital Español, Buenos Aires); Marcos Juan Zec & Pascual Valdez (Hospital General de Agudos Dalmacio Vélez Sársfield, Buenos Aires); Jorgelina Guyon, Ariel Chena (Hospital Lagomaggiore, Mendoza); Sergio Lasdica (Hospital Municipal Coronel Suárez, Buenos Aires); Martin Deheza, Schimdt Alejandra (Hospital General de Agudos Bernardino Rivadavia, Buenos Aires); Francisco Criado (Hospital Naval Puerto Belgrano, Bahía Blanca); Norma Beatriz Márquez (Policlínico Atlántico del Sur, Ríos Gallegos); Pablo Desmery & José Luis Scapellato (Sanatorio Anchorena, Buenos Aires); Gonzalo Javier Ríos & Cristian Casabella (Clínica Bazterrica, Buenos Aires)

Australia: Coordinators: Jasmin Board & Andrew Davies

Andrew Bersten, Elisha Matheson & Amy Waters (Flinders Medical Center, Adelaide); John Santamaria & Jennifer Holmes (St Vincent's Hospital, Melbourne); Cartan Costello, Manoj K Saxena & John Myburgh (St George Hospital, Sydney); Ellen Kinkel & Forbes McGain (The Western Hospital, Melbourne); Claire Cattigan & Allison Bone (Barwon Health, Geelong Hospital,

Geelong); Ian Seppelt, Leonie Weisbrodt & Cheryl Cuzner (Nepean Hospital, Sydney); Christopher MacIsaac, Deborah Barge & Tania Caf (Royal Melbourne Hospital, Melbourne); Cameron Knott & Graeme Duke (The Northern Hospital, Melbourne); Imogen Mitchell, Helen Rodgers, Rachel Whyte & Elisha Fulton (Canberra Hospital, Canberra); Jasmin Board, Andrew Davies & Alistair Nichol (Alfred Hospital, Melbourne); Hergen Buscher, Priya Nair & Claire Reynolds (St Vincent's Hospital, Sydney); Simon JG Hockley, Ian Moore & Katherine Davidson (Calvary Wakefield Hospital, Adelaide), David Milliss, Raju Pusapati & Helen Wong (Concord Hospital, Sydney); Jason Fletcher & Julie Smith (Bendigo Hospital, Bendigo); Paul Goldrick, Dianne Stephens & Jane Thomas (Royal Darwin Hospital, Darwin); Anders Aneman, Sutrisno Gunawan & Tom Cowlam (Liverpool Hospital, Liverpool); George Lukas & Rick McAllister (Royal Hobart Hospital, Hobart); Minka Springham, Joanne Sutton & Jeff Presneill (Mater Health Services, Brisbane); Tony Sutherland & Dianne Hill (Ballarat Health Services, Ballarat); Howard Connor, Jenny Dennett & Tim Coles (Central Gippsland Hospital, Sale).

Bolivia: Coordinator: Freddy Sandy

Sando Chavarria & Marcelo Choque (Hospital Obrero No.1, La Paz), Ronald Pairumani & Juan Guerra *(*Instituto Gastroenterológico, La Paz)

Brazil: Coordinator: Marco Antônio Soares Reis

José Carlos Versiani (Hospital Madre Teresa, Belo Horizonte); Eduardo Fonseca Sad (Hospital Luxemburgo , Belo Horizonte); Maria Aparecida Braga (Hospital Dia e Maternidade Unimed-BH; Belo Horizonte); Dinalva Aparecida Gomes (Hospital Vera Cruz, Belo Horizonte); Fernando Antônio Botoni (Hospital de Pronto Socorro Risoleta Tolentino Neves, Belo Horizonte); Maurício Meireles Góes (Hospital da Baleia, Belo Horizonte); Adriano José de Souza Teixeira (Hospital Belo Horizonte, Belo Horizonte); Frederico Costa Val Barros (Hospital da Polícia Militar, Belo Horizonte); Rogério de Castro Pereira (Hospital Felício Rocho, Belo Horizonte); Carlos Henrique Diniz Miranda (Hospital São Lucas, Belo Horizonte); Valério Trindade Lopes de Moura (Hospital Otaviano Neves – Medimig, Belo Horizonte); Hugo Urbano (Hospital Vila da Serra, Belo Horizonte); Valéria de Carvalho Magela (Hospital Santa Rita, Contagem); Lucas Viegas Martins (Hospital Unimed-BH, Belo Horizonte); Aline Camile Yehia (Hospital Júlia Kubitschek, Belo Horizonte); Bruno Bonaccorsi Fernandino (Hospital São Francisco-Setimig, Belo Horizonte); Marco Antônio Ribeiro Leão (Hospital São João de Deus, Divinópolis); Sérgio Azevedo Naves (Hospital São Lucas, Governador Valadares); Rovílson Lara (Hospital Arnaldo Gavazza Filho, Ponte Nova); Rovílson Lara (Hospital São João Batista, Viçosa); Rubens Altair Amaral de Pádua (Hospital Vaz Monteiro, Lavras); Janine Dias Alves (Santa Casa de Misericórdia de Ouro Preto, Ouro Preto); Aloísio Marques do Nascimento (Hospital Nossa Senhora das Graças, Sete Lagoas); Bruno do Valle Pinheiro (Hospital Universitário da Universidade Federal de Juiz de Fora, Juiz de Fora); Carlos Alberto Studart Gomes (Hospital de Messejana, Fortaleza); Marcelo Alcântara Holanda (Hospital Universitário Walter Cantídio, Fortaleza); Frederico Rodrigues Anselmo (Hospital Nossa Senhora Aparecida, Belo Horizonte).

Canada: Coordinator: Niall D. Ferguson

Neill Adhikari, Damon Scales, Robert Fowler, Cheromi Sittambalam, Mehar-Un-Nisa Raja & Nicole Marinoff (Sunnybrook Health Science Centre, Toronto Ontario); Lauralyn McIntryre, Shawna Reddie, Laura Jones, & Irene Watpool (Ottawa Hospital, Ottawa, Ontario); Jeffrey Singh, Andrea Matte, Marc Lipkus Madison Dennis & Ryan Albert (Toronto Western Hospital, Toronto, Ontario); Andrew Steel & Emily Stern (Toronto General Hospital, Toronto, Ontario); Michael Miletin, Antonio Raso, & Robyn Klages (William Osler Health Science Centre, Brampton, Ontario); Jan Friedrich, Orla Smith & Laura Wilson (St Michael's Hospital, Toronto, Ontario); Deborah Cook & Mark Bailey (St Joseph's Hospital, Hamilton, Ontario); Sangeeta Mehta, Stephen Lapinsky, Hannah Mathers, Cheryl Ethier, Stephanie Lubchansky & Samer Haj-Bakri (Mount Sinai Hospital, Toronto, Ontario); Dietrich Henzler, & Lisa Julien (Queen Elizabeth II Health Sciences Centre, Halifax, Nova Scotia).

Chile: Coordinator: Luis Soto-Román

Juan Carlos Maurelia (Hospital de Copiapo); César Antonio Maquilon (Instituto Nacional del Tórax, Santiago); Luis Soto-Germani (Hospital de Coquimbo).

China: Coordinator: Bin Du

Yan Kang & Bo Wang (West China Hospital, Chengdu); Fachun Zhou & Fang Xu (Chongqing Medical University 1st Hospital, Chongqing); Haibo Qiu & Yi Yang (Southeast University Zhongda Hospital; Nanjing); Qingyuan Zhan & Bing Sun (Beijing Chaoyang Hospital, Beijing); Zhenjie Hu & Bin Yu (Hebei Medical University 4th Hospital, Shijiazhuang); Xi Zhu & Yu Bai (Peking University 3rd Hospital; Beijing); Gang Li & Yi Li (Sino-Japanese Friendship Hospital, Beijing); Geng Zhang & Jianbiao Meng (Zhejiang Tongde Hospital; Hangzhou); Xiaobo Huang & Hong Pu (Sichuan Provincial Hospital; Chengdu); Bin Du & Daxing Yu (Peking Union Medical College Hospital, Beijing); Chuanyun Qian & Wei Zhang (Kunming Medical College 1st Hospital, Kunming); Yongjie Yin & Debiao Song (Jilin University 2nd Hospital, Changchun); Yunxuan Yue & Zhengxuan Lv (Kunming City 3rd People's Hospital, Kunming); Chengmin Yu & Qunmei Yao (Yunnan Chuxiong People's Hospital; Chuxiong); Xue Wang (Xi'an Jiaotong University 1st Hospital; Xi'an); Yuan Xu & Wei He (Beijing Tongren Hospital, Beijing); Mian Chen & Zhihua Hu (Hainan Medical College Hospital; Haikou); Dongpo Jiang & Jian Huang (Daping Hospital, Chongqing); Wei Yu (Yantai Yuhuangding Hospital; Yantai); Juanxian Gu (Zhejiang Haining People's Hospital, Naining); Yangong Chao (Beijing Huaxin Hospital, Beijing); Zhixiang Li (Fengrun District People's Hospital; Tangshan); Zhicheng Zhang (PLA Navy General Hospital, Beijing); Wanxia Li (Nanchang University 2nd Hospital, Nanchang); Zhenyang He (Hainan Provincial People's Hospital, Haikou); Jianguo Li & Chang Liu (Wuhan University Zhongnan Hospital, Wuhan); Tiehe Qin & Shouhong Wang (Guangdong General Hospital, Guangzhou); Feng Li (Nantong 1st People's Hospital, Nantong); Jun Jin & Jianhong Fu (Suzhou University 1st

Hospital, Suzhou); Hongyang Xu (Wuxi Municipal People's Hospital, Wuxi); Hongyuan Lin & Jianying Guo (PLA 304 Hospital, Beijing); Yalin Liu & Jinghua Wang (Beijing Hospital, Beijing); Maoqin Li & Jiaqiong Li (Xuzhou Central Hospital, Xuzhou); Lei Chen (Sun Yet-Sen University 6th Hospital, Guangzhou); Qing Song & Liang Pan (PLA General Hospital, Beijing); Xianyao Wan & Jiuzhi Zhang (Dalian Medical University 1st Hospital, Dalian); Weihai Yao & Yuhong Guo (Beijing TCM Hospital, Beijing)

Colombia: Coordinator: Marco González

Ricardo Buitrago (Clínica Shaio, Bogotá); Marcela Granados (Clínica Fundación Valle Lili, Cali); Guillermo Ortiz (Hospital Santa Clara, Bogotá); Cesar Enciso (Grupo Cimca Hospital San José, Bogotá); Mario Gómez (Grupo Cimca Hospital San José & Clínica Fundadores, Bogotá); Bladimir Alejandro Gil (Clínica Las Américas, Medellín); Juan Pablo Sedano, Luis Fernando Castro Castro (Centro Medico Imbanaco, Cali); Carlos Alberto Acosta (Hospital Federico Lleras Acosta, Ibague); Marco Gonzalez A.(Hospital San Rafael, Itagui); Francisco Molina (Clínica Universitaria Bolivariana, Medellín); Camilo Pizarro (Fundación cardiovascular colombiana, Bucaramanga); Mario Villabon (Grupo Cimca Hospital de Suba, Bogota); Carmelo Dueñas (Nuevo Hospital Bocagrande, Cartagena de Indias); Carlos Andrés Díaz (Hospital General de Medellín); Nelson Fonseca (Corbic, Medellín); Rubén Camargo (Clínica General del Norte, Barranquilla), Juan David Uribe (Clínica Cardiovascular, Medellín)

Denmark: Coordinator: Hans-Henrik Bülow

Simona Beniczky & Jens Brushoj (Naestved); Mikkel Præst & Henrik Guldager (Nykobing Falster); Birgitte Viebaek & Sine Wichman; (Roskilde); Anette Mortensen (Holbak); Susanne Andi Iversen (Slagelse); Bo Broberg & Line Museus Poulsen (Koge)

Dominican Republic: Edgard Luna (Hospital Universitario José María Cabral y Báez, Santiago)

Ecuador: Coordinator: Manuel Jibaja

Leonardo Pazmiño, Katty Trelles & Fabricio Picoita (Hospital Eugenio Espejo, Quito); Gustavo Paredes & Vanesa Ramírez (Hospital Enrique Garcés, Quito); Guillermo Falconí, Cristian Cevallos & Boris Villamagua; (Hospital Carlos Andrade Marín, Quito); Marco Escobar & Freddy Sánchez (Hospital de la Policía, Quito); Miguel Llano & Miguel Lazcano (Hospital General de las Fuerzas Armadas, Quito); Ramiro Puetate & José Miguel Guerrero (Hospital Pablo Arturo Suárez, Quito); Mijail Játiva & Myriam Montalvo (Hospital de los Valles, Quito); Franklin Villegas (Hospital Metropolitano, Quito); Luis González Zambrano, Ronnie Mantilla, Gina Quinde, Andrea Gimenez & Luis Gonzalez Mosquera (Hospital Luis Vernaza, Guayaquil); Henry Caballero & María Fernanda García (Hospital de SOLCA, Quito); Marcelo Ochoa, Soraya Puertas & Jackeline Coello (Hospital José Carrasco Arteaga, Cuenca); Opilio Córdova (Hospital Provincial Docente, Ambato); Mario Acosta (Hospital San Vicente de Paul, Ibarra).

Egypt: Medhat Soliman (Cairo University Hospitals, Cairo)

France: Coordinator: Arnaud W. Thille

Achille Kouatchet & Alain Mercat (CHU d'Angers); Laurent Brochard (CHU Henri Mondor, Créteil); François Collet (Centre Hospitalier De Saint-Malo); Guillaume Marcotte (Hôpital Édouard Herriot, Lyon); Pascal Beuret (Centre Hospitalier De Roanne); Jean-Christophe M. Richard, Gaëtan Bedunaeu, Pierre-Gildas Guitard & Fabien Soulis (CHU Charles Nicolle, Rouen); Frédéric Bellec (Centre Hospitalier De Montauban); Philippe Berger (Centre Hospitalier de Châlons en Champagne); Jack Richecoeur (Centre Hospitalier René Dubos, Pontoise); Dorothée Carpentier, Benoit Veber (CHU Charles Nicolle, Rouen); Salem Ould Zein, Géraldine Dessertaine (CHU De Grenoble); C. Canevet (Hôpital d'Armentières); Fabien Grelon (Centre Hospitalier Du Mans).

Germany: Coordinator: Konstantinos Raymondos

Rolf Dembinski & Rolf Rossaint (Universitaetsklinikum Aachen); Steffen Weber-Carstens (Charité Universitaetsklinikum, Berlin); Christian Putensen (Universitaetsklinikum Bonn); Maximillian Ragaller (Universitaetsklinikum Carl Gustav Carus, Dresden); Michael Quintel (Universitaetsklinikum der Georg-August-Universitaet, Goettingen); Winfried Schubert (Carl-Thiem-Klinikum Cottbus gGmbH); Herwig Gerlach & Arka Genaehr (Vivantes-Klinikum Neukoelln, Berlin); Thomas Bein & Heinrich Paulus (Klinikum der Universitaet Regensburg); Walter Brandt (Universitaetsklinikum, Magdeburg); Lutz Pfeiffer & Silke Frenzel (Hufeland Klinikum GmbH, Mühlhausen); Jens Geiseler (Asklepios Fachkliniken Muenchen-Gauting); Thoralf Kerner & P. Kruska (Allgemeines Krankenhaus Harburg, Hamburg); Leila Eckholt & Joachim Hartung (Vivantes Krankenhaus am Urban, Berlin-Kreuzberg); Oliver Jung (St.-Vincentius-Krankenhaus Speyer); Harald Fritz & Monika Holler (Staedtisches Krankenhaus Martha-Maria Halle-Doelau gGmbH); Johannes Busch & Andreas Viehöfer (Evangelisches Waldkrankenhaus Bad Godesberg gGmbH, Bonn); Jens Buettner (Evangelisches Krankenhaus Elisabethenstift gGmbH, Darmstadt); Jörn Schlechtweg & Achim Lunkeit & Roland Schneider (Klinikum Bad Salzungen GmbH); Maria Wussow & Nils Marquardt & Christian Frenkel (Staedtisches Klinikum Lueneburg); Sabine Meyer & Wolfram Wilhelm (St.-Marien-Hospital, Luenen); Falk Hildebrandt (Dietrich-Bonhoeffer Klinikum Neubrandenburg); Tumbass Volker & Thomas Lipp (Ermstalklinik Bad Urach); Cezar Mihailescu & Thomas Moellhoff (Katholische Stiftung Marienhospital Aachen); Thomas Steinke (Universitaetsklinikum der Martin-Luther-Universitaet Halle-Wittenberg); Oliver Franke & Marcus Ruecker (Lungenklinik Heckeshorn, Berlin); Markus Schappacher & Steffen Appel (Ermstalklinik Staedtisches Krankenhaus Sindelfingen); Heinz Kerger (Evangelisches Diakoniekrankenhaus, Freiburg); Andreas Schwartz (Bundeswehrkrankenhaus Ulm); Jan Dittmann & Jörg Haberkorn (Georgius-Agricola-Klinikum Zeitz); Wolfgang Baier (St. Nikolaus-Stiftshospital GmbH, Andernach); Hans-Friedrich Ginz (Kreiskrankenhaus Loerrach); Walter Seyde (Staedtisches Klinikum Wolfenbuettel)

Greece: Coordinator: Dimitros Matamis

Eleni Antoniadou (Gennimata Hospital, Thessaloniki); Pertsas Evangelos (Agios Pavlos General Hospital, Thessaloniki); Maria Giannakou (Ahepa Hospital, Thessaloniki).

Hungary: Zoltan Szentkereszty (Kenezy Hospital, Debrecen); Zsolt Molnar (University of Szeged)

India: Coordinator: Pravin Amin

Farhad N. Kapadia (Hinduja Hospital, Mumbai); Nagarajan Ramakrishnan (Apollo Hospitals, Chennai); Deepak Govil (Artemis Health Institute, Gurgaon Haryana);Anitha Shenoy & Umesh G (Kasturba Medical College, Manipal); Samir Sahu, (Kalinga Hospital, Odisha); Sheila Nainan Myatra (Tata Memorial Hospital, Mumbai); Subhash Kumar Todi (AMRI Hospitals Kolkata , West Bengal); Sanjay Dhanuka (CHL Apollo Hospital, Indore); Mayur Patel (Saifee Hospital, Mumbai); P Samaddar(Tata Main Hospital, Jamshedpur); Dhruva Chaudhry (PGIMS, Rohtak); Vivek Joshi & Srinivas Samavedam (CARE Hospitals ,Surat), Ankur Devendra Bhavsar (Spandan Multispeciality Hospital, Vadodar); Prachee Sathe (Ruby Hall Clinic , Pune); Sujoy Mukherjee (Calcutta medical research institute (CMRI), Kolkat)

Italy: Coordinator: Salvatore Maurizio Maggiore

Francesco Idone & Federica Antonicelli (Policlinico "Agostino Gemelli", Università Cattolica Del Sacro Cuore, Roma); Paolo Navalesi, Rosanna Vaschetto & Arianna Boggero (Ospedale Maggiore Della Carità, Università Del Piemonte Orientale "Amedeo Avogadro", Novara); Rosalba Tufano, Michele Iannuzzi & Edoardo De Robertis (Ospedale Policlinico "Federico II", Università Di Napoli, Napoli); Romano Tetamo & Andrea Neville Cracchiolo (Ospedale "Arnas Civico, Di Cristina, Benfratelli", Palermo), Antonio Braschi, Francesco Mojoli & Ilaria Curro' (ICU 1, Fondazione IRCCS Policlinico “S. Matteo”, Università Di Pavia, Pavia); Mirko Belliato, Chiara Verga & Marta Ferrari (ICU 2, Fondazione IRCCS Policlinico “S. Matteo”, Università Di Pavia, Pavia); Erika Mannelli, Valerio Mangani & Giorgio Tulli (Ospedale "San Giovanni Di Dio", Firenze); Francesca Frigieri & Armando Pedulla' (Ospedale "Santa Maria Annunziata", Firenze); Monica Rocco, Giorgia Citterio & A. Di Russo (Policlinico "Umberto I", Università La Sapienza, Roma); Gaetano Perchiazzi & Loredana Pitagora (Ospedale Policlinico, Università Di Bari, Bari); Antonio Pesenti & Michela Bombino (Ospedale "San Gerardo", Università Di Milano Bicocca, Monza); Davide Chiumello, Federica Tallarini & Serena Azzari (Fondazione IRCCS “Cà Grande” Ospedale Maggiore Policlinico, Università Di Milano, Milano); Antonina Pigna, Ivano Aprile & Marco Adversi (Policlinico Universitario "S. Orsola-Malpighi", Bologna); Antonio Corcione, Marianna Esposito & Annunziata Mattei (Ospedale "V. Monaldi", Napoli); Vito Marco Ranieri, Rosario Urbino & Ilaria Maria Mastromauro (Ospedale "San Giovanni Battista - Molinette", Università Di Torino, Torino); Antonino Giarratano, Maurizio Raineri Santi & Ambrogio Sansone (Policlinico "P. Giaccone", Università Di Palermo, Palermo)

Japan: Toru Katani (Tokyo Women's Medical University)

Korea: Coordinator: Younsuck Koh

Moo Suk Park (Hospital Severance, Yonsei University Health System, Seoul); Je Hyeong Kim (Hospital Korea University of Ansan); Kyung Chan Kim (Hospital Catholic University of Daegu); Hye Sook Choi (Hospital Dongguk University of Gyeongju); Yun Seong Kim (Hospital Pusan National University of Yangsan); Jin Hwa Lee (Hospital Ewha Womans University Mokdong, Seoul); Myung-Goo Lee (Hospital Chuncheon Sacred Heart Hospital, Hallym University Medical Center, Chuncheon); Won-Yeon Lee (Hospital Yonsei University Wonju Christian, Wonju); Jin Young An (Hospital Chungbuk National University, Chenogiu); Gee Young Suh (Samsung Medical Center, Sungkyunkwan University, Seoul); Ki-Suck Jung (Hallym University Medical Center, Anyang)

Mexico: Coordinator: Asisclo J Villagómez Ortiz

César Cruz Lozano (Hospital Regional de Pemex, Ciudad Madero); Zalatiel Maycotte Luna (Hospital Ángeles de las Lomas, México DF); José Francisco López Baca (Hospital Regional de Zona No.1 del IMSS, México DF); Víctor Manuel Sánchez Nava (Hospital San José TEC de Monterrey); Felipe Gilberto Vázquez de Anda (Centro Médico Nacional siglo XXI, IMSS, México DF)

Morocco: Coordinator: Amine Ali Zeggwagh

Tarek Dendane (Hôpital Ibn Sina, Rabat); Abderrahim Azzouzi (Hôpital Ibn Sina, Rabat); Ahmed Sbihi (Hôpital Ibn Sina, Rabat); Wajdi Maazouzi & Mourad Amor (Hôpital des Specialites, Rabat); Charki Haimeur (Hôpital Militaire D'Instruction Mohamed V, Rabat).

Netherlands: Coordinator: Michael A. Kuiper

Matty Koopmans (Medical Center Leeuwarden (MCL), Leeuwarden); Uli Strauch, Dennis Bergmans & Serge Heines (Universitair Medisch Centrum Maastricht, Maastricht); Sylvia den Boer (Spaarneziekenhuis, Hoofddorp); Bas M. Kors (Kennemer Gasthuis, Haarlem); Peter van der Voort (Onze Lieve Vrouwe Gasthuis (OLVG), Amsterdam); Paul J. Dennesen (Medisch Centrum Haaglanden, Den Haag); Bert Beishuizen, Ingrid van den Hul, Erna Alberts, Harry PPM Gelissen & Eduard Bootsma (Vrije Universiteit Medisch Centrum (VUMC), Amsterdam); Alexander Winsser & Roel Smit (Antonius Ziekenhuis, Sneek); Auke Reidinga (Tjongerschans Ziekenhuis, Heerenveen).

New Zealand: Coordinator: Jasmin Board & Andrew Davies

Kim Heus, Diane Mackle & Paul Young (Wellington Hospital, Wellington); Rachael Parke, Eileen Gilder & Jodi Brown (CVICU Auckland City Hospital, Auckland); Lynette Newby & Catherine Simmonds (DCCM Auckland City Hospital, Auckland); Jan

Mehrtens & Seton Henderson (Christchurch Hospital, Christchurch); Tony Williams, Judi Tai & Chantal Hogan (Middlemore, Auckland); Mary La Pine, John Durning & Sheree Gare (Waikato, Hamilton); Troy Browne, Shirley Nelson & Jennifer Goodson (Tauranga Hospital, Tauranga).

Panama: Julio Osorio (Hospital Rafael Hernández, Chiriquí)

Peru: Coordinator: Chabu Coronado

Rollin Roldán Mori (Hospital Edgardo Rebagliatti Martins, Lima); Rosa Luz López Martínez (Hospital Guillermo Almenara Irigoyen, Lima)

Poland: Adam Mikstacki & Barbara Tamowicz (Karol Marcinkowski University Of Medical Sciences, Poznan).

Portugal: Coordinator: Rui Moreno

Eduardo Almeida (Hospital Garcia de Orta, Almada); Joana Silvestre (Centro Hospitalar de Lisboa Central); Heloisa Castro, Irene Aragão & Susana Alves Ferreira (Centro Hospitalar Do Porto - Hospital Geral De Santo António); Nelson Barros (Centro Hospitalar Trás-Os-Montes E Alto Douro, Vila-Real); Filomena Faria (Ipo); Carlos André Correia Casado (Hospital Da Luz); Fausto Fialho Moura (Hospital de Cascais); Paulo Marcal (Hospital De São Sebastião); Ricardo Matos (Hospital de Santo Antonio dos Capuchos, Centro Hospitalar de Lisboa Central, E.P.E., Lisboa); António Alvarez (Centro Hospitalar de Lisboa Norte)

Russian Federation: Coordinator: Edward Nicolayenko

Mikhail Kirov (Hospital No.1, Arkhangelsk); Andrey Yaroshetskiy (Hospital No.7, Moscow); Andrei Piontek (Hospital No.14, Ekaterinburg); Valery Subbotin (National Institute Of Surgery Named After A.V. Vishnevskij, Moscow)

Saudi Arabia: Yaseen Arabi, Olivia Dulfo, Chafrina Marie Olay, Edgardo E. Tabhan (King Saud Bin Abdulaziz University for Health Sciences, Riyadh)

Spain: Coordinator: Nicolas Nin

Alfonso Muñoz & César Aragón (Hospital Carlos Haya, Málaga); Ana Villagrá (Corporación Sanitaria y Universitaria Parc Taulí, Sabadell); Ainhoa Rosselló, & Joan Maria Raurich (Hospital Universitario Son Espasses, Mallorca); María Garitacelaya, Miguel Ángel González-Gallego & Francisco Ortuño (Hospital Clínico Universitario San Carlos, Madrid); Miguel Fernández-Vivas (Hospital Virgen de la Arrixaca, Murcia); David Freire (Hospital Juan Canalejo, A Coruña); Francisco Guerrero & Francisco Manzano (Hospital Virgen de las Nieves, Granada); Juan Carlos Sotillo (Hospital Universitario Gregorio Marañón, Madrid); Alejandra Bustos (Hospital de Torrevieja); Alfredo Padrón, Pedro Rosas, Rafael Morales & Liliana Caipe (Hospital Doctor Negrín, Las Palmas de Gran Canaria); Maurizio Bottiroli (Hospital de la Santa Creu i Sant Pau, Barcelona); José María Nicolás (Hospital

Clinic-IDIBAPS, Barcelona); Marta Ugalde (Hospital de Cruces, Barakaldo); Javier Ruiz (Hospital Sagrado Corazón, Barcelona); Lucia Capilla (Hospital Morales Meseguer, Murcia); Guillermo Muñiz (Hospital Central de Asturias, Oviedo); Jesús Sánchez-Ruiz (Hospital General de Jerez de la Frontera); Javier Cebrián, Begoña Balerdi, Elena Parreño & Alvaro van Bommel (Hospital Universitario La Fe, Valencia); César Pérez-Calvo (Fundación Jiménez Díaz, Madrid); Irene Dot (Hospital del Mar, Barcelona); Javier Blanco (Complejo Hospitalario de Ciudad Real); Raquel Manzanedo & José J. Blanco (Hospital Insular de Gran Canaria); Daniel Fontaneda, Raúl González & Javier Díaz Domínguez (Complejo Hospitalario de León); Alfonso Moreno (Hospital San Pedro. Logroño); Antonio Reyes & Ian Carrasco (Hospital de la Princesa, Madrid); Itziar Mintegui, Rosa Sebastián & Javier García-Alonso, (Complejo Hospitalario de Donostia); Carolina Lorencio & Josep Maria Sirvent (Hospital Universitario Dr.Josep Trueta, Girona); Patricia Jimeno (Hospital General de Segovia); Miguel León (Hospital Arnau de Vilanova, Lleida); Pedro Galdos (Hospital Universitario Puerta de Hierro, Majadahonda); Nuria Alonso (Hospital Universitario Nuestra Santa María del Rosell, Cartagena); Julia López-Díaz (Hospital Universitario La Paz, Madrid); María Victoria de la Torre, Jorge Vidal Hernández & Nicolás Zamboschi (Hospital Universitario Virgen de la Victoria, Málaga); Francisco Lucena (Hospital Universitario de Valme, Sevilla); Gemma Rialp (Hospital Son Llatzer, Mallorca); Raquel Montoiro (Hospital Clínico Lozano Blesa, Zaragoza); Victoria Goñi, María Ángeles Pena & Antonio Maestre (Hospital Virgen del Rocío, Sevilla); Marc Fabra, Jacinto Baena & Eva Benveniste (Hospital Germans Trias I Pujol, Badalona); Susana Temprano (Hospital 12 de Octubre, Madrid); Jesús Sánchez (Hospital de Rio Hortega, Valladolid); Carmen Campos (Hospital Universitario Dr.Peset, Valencia); Sara Cabañes (Hospital Santiago Apóstol, Vitoria); María Elena Pérez-Losada & José Claudio Leo (Complejo Hospitalario de Salamanca); Enrique Piacentini (Hospital Mutua de Terrassa); María del Carmen de la Torre (Hospital de Mataró); Laura Álvarez-Montero, Fernando Sánchez (Hospital Xeral Calde, Lugo); Antonio Viñuales (Hospital Lluis Alcanyis, Xàtiva); Bernabé Álvarez (Hospital General de Alicante); Javier Castañeda (Hospital Clínico de Valladolid); Ángela Alonso (Hospital de Fuenlabrada); María Isabel Ruiz (Complejo Hospitalario de Jaén); Pedro Jesús Domínguez (Hospital Juan Ramón Jiménez, Huelva); Marcos Delgado (Complejo Hospitalari de Manresa); Eugenio Palazón (Hospital Universitario Reina Sofía, Murcia); Antonio García-Jiménez (Hospital Arquitecto Marcide, Ferrol); Rosa Álvaro (Hospital de La Plana, Castellón); Clara Laplaza, Eva Regidor & Enrique Maraví (Complejo Hospitalario de Navarra, Pamplona); José María Quiroga (Hospital de Cabueñes, Gijón); Amalia Martínez de la Gandara (Hospital Infanta Leonor, Madrid); Cecilia Carbayo (Hospital Torrecárdenas, Almería); María Luisa Navarrete (Hospital San Juan, Alicante); Manuel Valledor & Raquel Yano (Hospital San Agustín, Avilés); José María Gutiérrez (Hospital General de Albacete); Javier González-Robledo (Hospital Clínico de Salamanca); Amparo Ferrandiz, Alberto Belenguer & Lidón Mateu (Hospital General de Castellón); Laura Sayagues (Complejo Hospitalario de Santiago de Compostela); María José Tolón (Hospital Royo Vilanova, Zaragoza); Nieves Franco (Hospital de Móstoles); Elena Gallego (Hospital San Pedro de Alcántara, Cáceres); Félix Lacoma (Hospital Quirón, Madrid); Patricia Albert (Hospital del Sureste, Arganda); Vicente Arraez (Hospital Universitario General, Elche); Mar Gobernado (Hospital General de Soria); Susana Moradillo (Hospital Río Carrión, Palencia); Carolina Gímenez-Esparza (Hospital de la Vega

Baja, Orihuela); Teresa Sánchez de Dios (Complejo Hospitalario Montecelo, Pontevedra); Carlos Marian Crespo (Hospital General de Guadalajara); Cecilia Hermosa, Federico Gordo (Hospital del Henares, Coslada); Genis Carrasco (Hospital SCIAS, Barcelona); María Ángeles Alonso (Trauma ICU, Hospital 12 de Octubre, Madrid); Alejandro Algora (Fundación Hospital Universitario de Alcorcón, Madrid); Raúl de Pablo (Hospital Príncipe de Asturias, Alcalá de Henares); Sofía García (Hospital del Poniente, El Ejido); Ana Carolina Caballero (Hospital de Zamora); José María Montón (Hospital Obispo Polanco, Teruel); Teresa Mut (Hospital Provincial de Castellón); Eva Manteiga (Hospital Infanta Cristina, Parla); Alejandro de la Serna (Hospital de Galdakao); Ana Esther Trujillo (Hospital General de La Palma); Rafael Blancas (Hospital del Tajo, Aranjuez); Inmaculada Vallverdú (Hospital Universitario San Juan, Reus); José Manuel Serrano (Hospital Universitario Reina Sofía, Córdoba); Miquel Ferrer (Hospital Clinic-IDIBAPS, Barcelona); Juan Diego Jiménez (Hospital de Don Benito); Carlos Gallego (Hospital Infanta Elena, Valdemoro); Dolores Vila (Hospital Meixoeiro, Vigo); Luis Marina (Complejo Hospitalario de Toledo); Valentín Parra (Hospital de Sagunto); Juan Ramón Cortés (Complejo Hospitalario de Ourense).

Taiwan: Chen Chin-Ming & Ai-Chin Cheng (Chi Mei Medical Center, Tainan City)

Tunisia: Coordinator: Fekri Abroug

Besbes Mohamed (Hospital Abderrahmane Mami, Ariana); Imed Chouchene (Hospital Universitarie Farhat Hached, Sousse); Mounir Bouaziz (CHU Habib Bourguiba, Sfax); Stambouli Neji & Islem Ouanes (Hospital Fattouma Bourguina, Monastir); Ayed Samia (Hospital Taher Sfar City :Mahdia); Mustapha Ferjani (Military Hospital Tunis)

Turkey: Coordinator: Nahit Cakar

Ismail Kati (Medical Faculty of Yuzuncu Yil University, Van) Ali Aydım Altunkan (Faculty of Medicine Mersin University); Remzi Iscimen (Uludag University Faculty of Medicine, Bursa); Zafer Dogan (Sutcu İmam University, Kahramanmaras); Bilge Çetin(Erciye Üniversity); Tayfun Adanir (Atatürk Hospital , Izmir); Sabriye Guvenc (Anadolu Medical Center Istanbul) Unase Büyükkoçak (Kirikkale University).

United States: Coordinator: Antonio Anzueto

Ashley Ellis & Gary Kinasewitz (Oklahoma University Health Science Center &VA medical Center, Oklahoma City); Allan Walkey & Phil Alkana; (Boston Medical Center, Boston); Gregory A. Schmidt, Susan Gillen, Kathleen Lilli, Jennifer Twombley, Denice Wells & Larry Welder; (University of Iowa Hospitals and Clinics, Iowa Vity); Alejandro Arroliga, Alfredo Vasquez-Sandoval, Vincent John Scott, Craig Cernosek & Christopher Spradley; (Temple Clinic, Scott & White Healthcare, Temple); Dimple Tejwani, Sindhaghatta Venkatram & Gilda Diaz-Fuentes (Bronx Lebanon Hospital Center, New York); Amber Monson, Anthony Saleh, Madhav Gudi & George Liziamma; (New York Methodist Hospital, New York); Mohamed A Saad, Crissie De Spirito, Bryan Beatty, Samir Vermani & Crissie Despirito; (University of Louisville School of Medicine Hospital, Louisville);

Zaza Cohen, Amee Patrawalla, Samir Abdelhadi, Rupesh Vakil & Steven Y. Chang (UMDNJ-New Jersey Medical School, Newark); Brian Sherman, Rosanna Del Giudice & John Oropell (The Mount Sinai Medical Center, New York); Timothy D Girard, Cayce Strength, Joyce Okahashi, Leanne Boehm & Matthew Kirchner (Vanderbilt University School Of Medicine, Nashville); Ashley Ellis & Gary Kinasewitz (Oklahoma City VA Medical Center, Oklahoma City); Erwin J. Oei, Sebastian Circo, Nelson Medina & Mohammed Al-Jagbeer (Morristown Medical Center, Morristown); V.J. Cardenas, Jr & Smyth Smith (University Of Texas Medical Branch at Galveston); Shelby Sutton, Marcela Canola-Mazo, Tim Houlihan, Yogeet Kaur & Travis Parry (University Hospital San Antonio and South Texas Veterans Health Care System); Craig A. Piquette & Kerry Canady (Omaha VA Medical Center. Omaha); Rahul Nanchal & Dana K. Soetaert (Medical College Of Wisconsin); Maria del Mar Torres-Perez, Carlos Robles-Arias & William Rodriguez-Cintron (VA Caribbean Health Care System, San Juan, Puerto Rico); Mark Tidswell, Jennifer Germain, Lori-Ann Kozikowski & Erin Braden (Baystate Medical Center, Springfield); Geneva Tatem (Henry Ford Hospital Detroit)

Uruguay: Coordinator: Javier Hurtado

Alberto Deicas (CASMU Nº2, Montevideo); Daniel Weiss (Hospital Pasteur, Montevideo); Marta Beron (Hospital Maciel, Montevideo); Román Garrido (Hospital Evangélico, Montevideo); Cristina Santos & Mario Cancela (Hospital de Clínicas, Montevideo); Raúl Lombardi (Impasa, Montevideo); Pedro Alzugaray (CAAMOC, Carmelo, Sanatorio Americano, Montevideo & Orameco, Colonia); Jorge Gerez (Hospital Policial, Montevideo); Silvia Mareque (Sanatorio CAMS, Mercedes); Graciela Franca (Circulo Católico, Montevideo); Oscar Cluzet (Sanatorio Americano, Montevideo); Edgardo Nuñez (Sanatorio Mautone & Hospital de Maldonado, Maldonado); Julio Pontet (Hospital de Florida, Florida); Sergio Cáceres (Centro Cardiológico Sanatorio Americano, Montevideo); Elias Caragna (CRAMI, Las Piedras); Alberto Soler (COMEPA & Hospital Escuela del Litoral, Paysandú); Frank Torres (Sanatorio Cantegril, Punta del Este); Gastón Pittini (CAAMEPA, Pando).

Venezuela: Coordinator: Gabriel d`Empaire

Stevens Salva & Fernando Pérez (Hospital de Clínicas de Caracas); Clara Pacheco & Zoraida Parra (Hospital Clínico Universitario de Caracas); Ingrid Von der Osten (Hospital Miguel Pérez Carreño, Caracas); Luis Williams & José Salinas (Hospital Centro de Especialidades de Anzoategui); Eutimio Pacheco & Pablo Carpio (Hospital José María Vargas, La Guaira)

Vietnam: Do Danh Quynh, Pham Thi Van Anh, Nguyen Huu Hoang & Nghuyen ba Tuan (Viet Duc Hospital, Hanoi).
